# Survival in Men Treated for Lung Cancer: A Single-Center Retrospective Cohort Study in Poland

**DOI:** 10.3390/healthcare14070970

**Published:** 2026-04-07

**Authors:** Magdalena Królikowska-Jerużalska, Magdalena Kurkiewicz, Aleksandra Moździerz, Anna Rzepecka-Stojko, Jerzy Stojko

**Affiliations:** 1 Department of Toxicology, Toxicological Analysis and Bioanalysis, Faculty of Pharmaceutical Sciences in Sosnowiec, Medical University of Silesia in Katowice, 41-200 Sosnowiec, Poland; 2 Faculty of Medicine, Branch in Bielsko-Biała Medical University of Silesia in Katowice, 43-382 Bielsko-Biała, Poland; 3 Department of Drug and Cosmetics Technology, Faculty of Pharmaceutical Sciences in Sosnowiec, Medical University of Silesia in Katowice, 41-200 Sosnowiec, Poland

**Keywords:** lung neoplasms, antineoplastic protocols, neoadjuvant therapy, male patients, survival

## Abstract

**Highlights:**

**What are the main findings?**

- Analysis of 1431 men showed that early diagnosis and surgical resection significantly improve survival in both SCLC and NSCLC patients.

**What are the implications of the main findings?**

- The results underscore the critical need for lung cancer screening programs to detect the disease at operable stages.

**Abstract:**

**Introduction:** Lung cancer remains the leading cause of cancer-related mortality among men in Poland. Prognosis is generally poor, largely due to late diagnosis at advanced stages and the aggressive biological nature of the disease. **Aim:** This study aimed to evaluate the effectiveness of various treatment modalities and determine their impact on overall survival in male patients diagnosed with small-cell (SCLC) and non-small-cell lung cancer (NSCLC). **Methods:** This retrospective cohort study analyzed 1431 men (mean age: 61.5 years) treated at the Katowice Oncology Center in Poland between 2002 and 2012. Overall survival was assessed using the Kaplan–Meier method and multivariable Cox proportional hazards regression. Evaluated prognostic factors included clinical stage, surgical intervention (partial or total lung resection), first-line treatment regimen, and the number of treatment cycles. **Results:** Survival probabilities declined progressively with advancing clinical stage for both SCLC and NSCLC. Patients who underwent surgical resection demonstrated significantly longer survival compared to non-surgically treated patients (*p* < 0.001). Furthermore, combined radiochemotherapy yielded superior therapeutic outcomes compared to chemotherapy alone. In the non-surgical NSCLC cohort, first-line treatment with platinum derivatives combined with gemcitabine resulted in the highest 1-year survival rate compared to other pharmacological schemes. **Discussion:** The high mortality observed within the first 12 months post diagnosis reflects the late-stage presentation common during the study period. The findings align with established oncological principles, confirming that surgical resection and multimodal therapies offer the greatest survival advantages for eligible patients. **Conclusions:** Survival rates for both SCLC and NSCLC are overwhelmingly dictated by early diagnosis and the feasibility of surgical resection. Improving long-term outcomes depends heavily on implementing effective lung cancer screening programs to detect the disease at operable stages and utilizing optimized combined treatment protocols.

## 1. Introduction

Lung cancer is the second most commonly diagnosed malignancy worldwide and in Poland, remaining a major oncological challenge due to its high incidence and poor treatment outcomes. While incidence and mortality rates have recently shown an increasing trend among women, a decreasing trend has been observed among men [1]. The disease is broadly classified into non-small-cell lung cancer (NSCLC), which accounts for approximately 80–85% of diagnoses, and small-cell lung cancer (SCLC), representing about 15% of cases. It remains the leading cause of cancer-related mortality in Poland, with active and passive exposure to tobacco smoke being the most significant risk factor, responsible for 85–90% of all cases [2,3].

### 1.1. The Situation in Poland

In Poland, the diagnostic pathway typically begins with a general practitioner referring suspected cases to hospital pulmonology departments. While computed tomography (CT) is widely utilized (approximately 88% of cases), positron emission tomography (PET) is performed in only 36% of patients [4]. Furthermore, access to advanced biomarker-driven diagnostics, such as next-generation sequencing (NGS), remains remarkably low due to insufficient reimbursement and limited systemic access, posing a significant challenge for lung cancer patients [5].

### 1.2. Study Objectives

Given this epidemiological context and the diagnostic landscape in Poland, this study aimed to evaluate the effectiveness of various lung cancer treatment modalities and their impact on overall survival among male patients. The analysis was based on historical data from patients treated at the Stanisław Leszczyński Hospital in Katowice (currently the Katowice Oncology Center) between 2002 and 2012.

### 1.3. Gene Mutations Associated with Lung Cancer

Contemporary lung cancer diagnostics and treatment planning are increasingly guided by molecular profiling. Key genetic alterations, along with their typical clinical and demographic characteristics and associated targeted therapies, are summarized in Table 1.

## 2. Materials and Methods

An analysis of patient outcomes was conducted among individuals treated for malignant lung tumors at the Stanisław Leszczyński Hospital in Katowice (currently the Katowice Oncology Center, KCO), between 2 January 2002 and 31 December 2012. The initial stage of the study involved the creation of a patient registry based on archived hospital medical records, including all patients diagnosed with malignant lung neoplasms coded as C34 according to the International Statistical Classification of Diseases and Related Health Problems (ICD).

### 2.1. Study Design and Setting

This was a retrospective, single-center observational cohort study evaluating the survival of male patients treated for lung cancer at the Stanisław Leszczyński Hospital in Katowice (currently the Katowice Oncology Center, KCO). The study period covered patients treated between 2 January 2002, and 31 December 2012. This specific timeframe was chosen to ensure a sufficiently long follow-up period (allowing for 5- and 10-year survival analyses) and to evaluate the efficacy of standard chemotherapy and radiochemotherapy protocols administered before the widespread introduction and reimbursement of modern immunotherapy and targeted biological agents in Poland. The findings are reported in accordance with the STROBE (Strengthening the Reporting of Observational Studies in Epidemiology) guidelines [15]; we emphasize that these guidelines were followed strictly for the reporting of our findings, rather than for the conduct of the study.

### 2.2. Participants: Inclusion and Exclusion Criteria

The initial study population was identified by retrospectively searching the hospital’s archival medical records for consecutive male patients diagnosed with malignant lung neoplasms, coded as C34 according to the International Statistical Classification of Diseases and Related Health Problems (ICD-10). To be included in the final analysis, patients had to meet the following inclusion criteria:

- Male sex;
- Age 18 years or older;
- Histopathologically confirmed primary lung cancer (SCLC or NSCLC);
- Receipt of at least one cycle of oncological treatment (chemotherapy, radiotherapy, or surgery) at the KCO during the specified timeframe.

Patients were excluded from the study if they met any of the following criteria:

- Diagnosis of a secondary (metastatic) lung tumor from another primary site;
- Incomplete essential medical documentation that precluded the determination of the clinical stage or the exact treatment regimen administered;
- Loss to follow-up preventing the determination of the date of death or survival status.

After applying these criteria, a final cohort of 1431 consecutive patients was included in the statistical analysis.

### 2.3. Data Sources and Variables

Data were manually extracted from archival paper and electronic hospital medical records. The primary outcome variable was Overall Survival (OS), defined as the time from the date of histopathological diagnosis to the date of death from any cause. Information regarding the dates of death was obtained by submitting official written requests to the relevant municipal civil registries; patient residency details and registry office addresses were verified using standard internet search engines.

The following clinicopathological variables were extracted for analysis: patient age, histopathological diagnosis, clinical stage, extent of surgical intervention (partial or total lung resection), the specific treatment regimen used in the first cycle, and the total number of treatment cycles administered (up to the third cycle; patients receiving more than three cycles were grouped together).

Because clinical stage according to the TNM classification was not explicitly recorded in all historical medical charts (or predated the expanded TNM classification for SCLC), staging was retrospectively determined or standardized by the investigators based on available clinical data, including radiographic imaging, computed tomography (CT) reports, and histopathological descriptions.

### 2.4. Statistical Analysis

Initial data collection and verification were performed using Microsoft Excel 2010. All subsequent statistical analyses and graphical presentations were generated using STATISTICA version 12.5 (StatSoft). Overall survival (OS) probabilities were estimated using the Kaplan–Meier method, which is well-suited for analyzing follow-up periods of varying lengths. Differences in survival curves between patient subgroups were evaluated using the log-rank test. Furthermore, to assess the independent impact of multiple prognostic factors simultaneously (such as clinical stage, surgical resection, treatment regimen, and number of cycles), a multivariable Cox proportional hazards regression model was utilized. All tests were two-sided, and a *p*-value of <0.05 was considered to indicate statistical significance.

## 3. Results

### 3.1. Study Population Characteristics

The study group consisted of 1431 men aged 28 to 82 years, with a mean age of 61.5 years. The largest proportion of patients (58%) were aged between 55 and 69 years. Patients younger than 45 years accounted for only 3% of the study population, while those aged 75 years and older constituted approximately 6%. During data collection, histopathological tumor diagnoses recorded in the archived medical documentation were evaluated. Due to the retrospective nature of the analysis, histopathological classification was not always precisely specified or amenable to verification. Consequently, in the final stage of analysis, patients were divided into two main groups: those diagnosed with small-cell lung cancer (SCLC; 430 patients, 30%) and those diagnosed with non-small-cell lung cancer (NSCLC; 1001 patients, 70%). Within the NSCLC group, squamous-cell carcinoma was diagnosed in 612 patients (43%), adenocarcinoma in 196 patients (14%), and a subgroup designated as “other” for the purposes of this study included 150 patients (10%) with unspecified NSCLC, 31 patients (2%) diagnosed with lung carcinoma not otherwise specified, and 12 patients (1%) with large-cell carcinoma. Due to their relatively small numbers, these latter categories were combined into a single group for statistical analysis.

Table 2 presents the age distribution according to the histological type of lung cancer. Among the 430 patients diagnosed with small-cell lung cancer (SCLC), patient ages ranged from 34 to 82 years, with a mean age of 61.6 years and a median of 62 years. In the group of 612 patients with squamous-cell carcinoma, the mean age was 62.1 years, with a median of 63 years and ages ranging from 37 to 81 years. Among the 196 patients diagnosed with adenocarcinoma, the mean age was 60.2 years, with a median of 61 years and ages ranging from 28 to 78 years. In the final group of 193 patients with other histological subtypes, the mean age was 60.8 years and the median age was 60 years, with patient ages ranging from 35 to 80 years. Among the above-mentioned histological subgroups, patients with adenocarcinoma demonstrated the highest proportion of individuals younger than 45 years of age (5.1%) and the lowest proportion of patients aged 75 years and older (2.6%).

Clinical stage according to the TNM classification was not explicitly recorded in all medical records. Therefore, staging was retrospectively determined based on available data, including radiographic and computed tomography reports as well as histopathological diagnoses. A similar situation applied to patients treated for small-cell lung cancer prior to 2009, when the TNM classification was expanded to include SCLC. In the study population, the following distribution of clinical stages at the time of diagnosis was observed: stage IA—23 patients, IB—25 patients, IIA—103 patients, IIB—115 patients, IIIA—363 patients, IIIB—296 patients, and IV—506 patients (Table 3).

In the entire study population, 19% of patients were diagnosed at clinical stages I and II. The highest proportion of patients at these early stages—over 27%—was observed among those diagnosed with adenocarcinoma.

### 3.2. Treatment Characteristics

A total of 188 patients (13.1% of the study population) underwent surgical resection, including resection of one pulmonary lobe, two lobes, or the entire lung. Lobectomy was performed in 133 patients, bilobectomy in 19 patients, pneumonectomy in 35 patients, and one patient underwent lingulectomy. These surgical procedures were not performed at the Stanisław Leszczyński Hospital in Katowice (currently the Katowice Oncology Center, KCO). Among all patients, surgical procedures involving partial or total lung resection were most frequently performed in those with stage I disease, where as many as 50% of patients underwent surgery. In stage II disease, surgical treatment was applied in 43% of patients, in stage III in 9%, and in stage IV in slightly over 2%. Patients diagnosed with adenocarcinoma underwent surgical treatment most often, with 34% of patients in this subgroup receiving lung resection.

Due to the extensive size of the collected dataset, platinum-based agents—cisplatin and carboplatin—were analyzed jointly for the purposes of this study and classified as platinum derivatives (PL). Both platinum analogs are used in the treatment of small-cell and non-small-cell lung cancer. Cisplatin is a highly toxic agent associated with numerous adverse effects, including nausea, vomiting, neurotoxicity, nephrotoxicity, ototoxicity, and bone marrow suppression [16]. Carboplatin exhibits an identical mechanism of action; however, its antitumor activity and toxicity profile differ from those of cisplatin. It is considered less toxic, with myelosuppression being its most significant adverse effect. The choice of treatment regimen and therapeutic intent (radical vs. palliative) was always determined by the physician based on the patient’s clinical evaluation, taking into account factors such as age, renal function, cardiovascular performance, bone marrow reserve, and comorbidities including diabetes mellitus [17]. For statistical analysis, the first three cycles of administered treatment were included in the analysis. Patients who received more than three treatment cycles were grouped together, representing approximately 3% of the study population (Table 4).

After analyzing the treatment regimen data, patients were categorized according to the chemotherapy protocol used in the first treatment cycle (Table 5). We attempted to analyze subsequent cycles; however, the large number of small subgroups precluded meaningful statistical analysis. In the cohort of patients with small-cell lung cancer (SCLC), four different chemotherapy regimens were administered in the first cycle. The largest subgroup (411 patients, 96%), treated with a platinum derivative (cisplatin or carboplatin) in combination with etoposide, was selected for further analysis.

Among patients with non-small-cell lung cancer (NSCLC), 13 different regimens were used in the first cycle. Five of the largest subgroups were included in further analysis:

• Platinum derivative (cisplatin or carboplatin) with vinorelbine (525 patients, 52%);
• Platinum derivative (cisplatin or carboplatin) with gemcitabine (262 patients, 26%);
• Platinum derivative (cisplatin or carboplatin) with etoposide (82 patients, 8%);
• Gemcitabine monotherapy (60 patients, 6%);
• Vinorelbine monotherapy (47 patients, 5%).

In reporting the treatment regimens used, the analysis was based on the active pharmaceutical ingredient rather than the commercial trade name. This approach was necessary because the hospital procured medications through public tenders, which resulted in frequent brand changes for the same active substances over the 10-year study period.

Mortality data for the study population were obtained directly from the relevant municipal civil registries. Patient residency details and registry office addresses were verified using standard internet search engines, resulting in a total of 32 formal requests sent to various registries. The effectiveness of the treatment modalities was assessed based on overall survival (OS).

Data were initially collected and verified using Microsoft Excel 2010. Patient subgroups were created in Excel and subsequently imported into STATISTICA version 12.5 (StatSoft) for statistical analysis and graphical presentation. Overall survival was estimated using the Kaplan–Meier method, which appropriately accommodates follow-up periods of varying lengths—a crucial consideration in oncology research. Survival curves were compared using the log-rank test, with statistical significance set at *p* < 0.05. This allowed for the comparison of survival times across several key variables, including:

- Histopathological diagnosis;
- Clinical stage at diagnosis;
- Surgical resection status;
- Extent of treatment (number of completed chemotherapy and radiotherapy cycles);
- First-line treatment regimen.

In addition to these primary variables, demographic factors such as age were also evaluated. To simultaneously assess the independent impact of multiple prognostic factors on patient survival, multivariable Cox proportional hazards regression analysis was performed. The results are presented in tabular form.

### 3.3. Analyses of Survival Probability

Detailed analyses regarding survival across different age groups, specific surgical subtypes, and outcomes for other chemotherapy regimens not discussed in the main text are available in the Supplementary Materials (Figures S1–S28).

Overall survival was evaluated in a cohort of 1431 patients with non-small-cell lung cancer (NSCLC) and small-cell lung cancer (SCLC) using the Kaplan–Meier method. In both groups, the most pronounced decline in survival probability occurred during the first 12 months after diagnosis, regardless of patient age. After approximately 60 months, the survival curves reached a low plateau, reflecting the high long-term mortality associated with both lung cancer subtypes.

**Figure 1:**
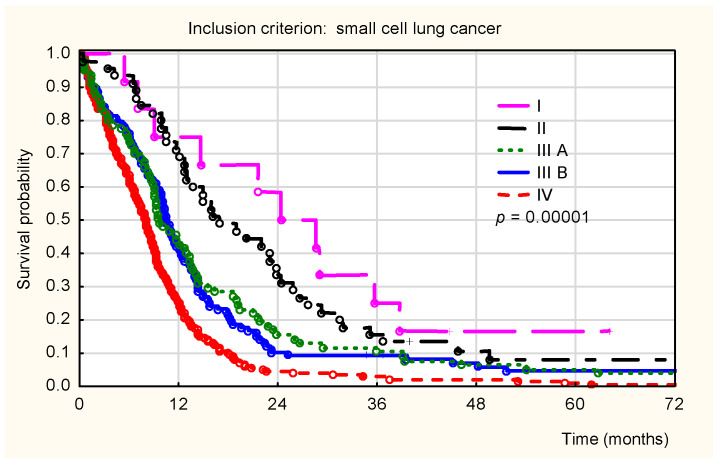
Kaplan–Meier overall survival curves for patients diagnosed with small-cell lung cancer (SCLC) stratified by clinical stage. The lines represent the following: pink solid line (Stage I), black solid line (Stage II), green dotted line (Stage IIIA), blue dash-dotted line (Stage IIIB), and red dashed line (Stage IV). Significant differences were observed between stages (*p* < 0.001). Stages I–IV were defined according to the TNM classification (8th edition).

For SCLC patients, overall survival differed significantly by clinical stage (*p* < 0.001; Figure 1). These differences were observed mainly between 12 and 36 months after diagnosis. As expected, the highest survival probability was noted in patients diagnosed with stage I disease, whereas the lowest was observed in those with stage IV disease.

**Figure 2:**
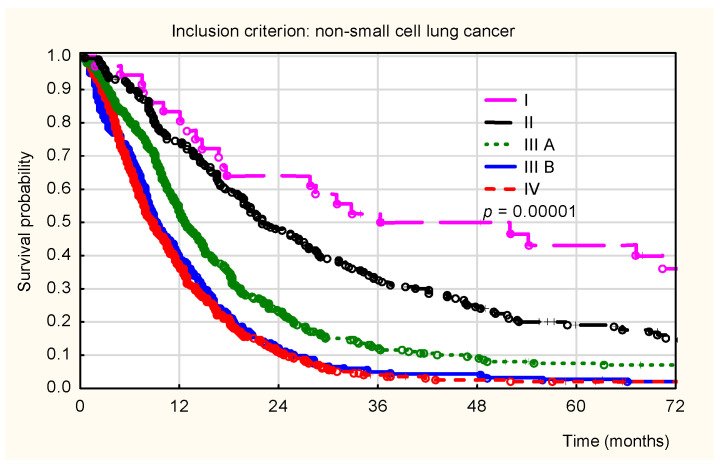
Kaplan–Meier overall survival curves for patients diagnosed with non-small-cell lung cancer (NSCLC) stratified by clinical stage. Legend: pink solid line (Stage I), black solid line (Stage II), green dotted line (Stage IIIA), blue solid line (Stage IIIB), and red dashed line (Stage IV). Statistical significance is indicated by *p* < 0.001. Stages I–IV were defined according to the TNM classification (8th edition).

For NSCLC patients, overall survival differed significantly depending on the clinical stage (*p* < 0.001; Figure 2). The longest survival was observed in patients with stage I disease, whereas the shortest survival was noted in those with stages IIIA, IIIB, and IV disease. These survival differences remained clearly evident throughout the entire follow-up period.

Among surgically treated patients, overall survival did not differ significantly between NSCLC and SCLC subtypes (log-rank test, *p* = 0.989). Throughout the entire follow-up period, the survival curves demonstrated a comparable course, with a gradual decline in survival probability observed in both groups. No distinct time intervals with divergent survival patterns were identified, indicating similar long-term outcomes in surgically treated patients regardless of lung cancer histology.

Similarly, for patients receiving non-surgical treatment, overall survival was comparable between the NSCLC and SCLC groups (log-rank test, *p* = 0.067). Both survival curves showed a steep decline in survival probability during the initial period of follow-up, followed by a plateau at low survival levels in later time intervals. Although patients with non-small-cell lung cancer tended to exhibit slightly higher survival probabilities throughout most of the observation period, this trend did not reach statistical significance.

In the SCLC cohort, overall survival was analyzed based on the applied treatment modality. Survival curves were generated for patients receiving surgical treatment and those managed with non-surgical approaches. The analysis revealed a statistically significant difference in overall survival between the treatment groups (log-rank test, *p* = 0.001). Patients treated surgically demonstrated markedly longer overall survival compared with those receiving non-surgical treatment. The divergence between survival curves was evident early after treatment initiation and persisted throughout the entire follow-up period, indicating a sustained survival benefit associated with surgical management in this subgroup.

**Figure 3:**
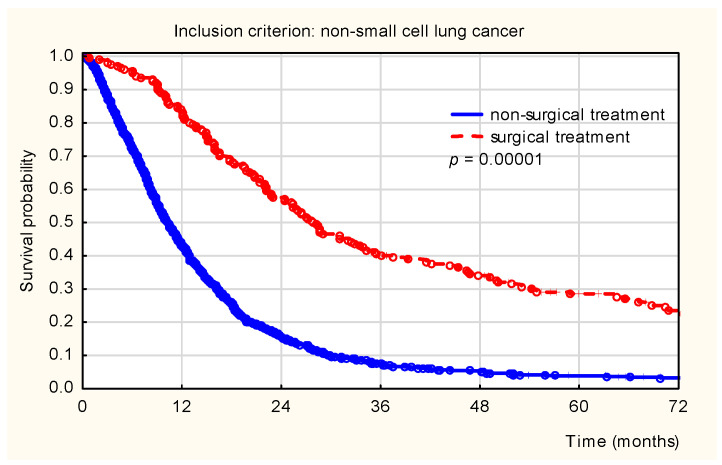
Kaplan–Meier overall survival curves for patients diagnosed with non-small-cell lung cancer (NSCLC), stratified by surgical versus non-surgical treatment.

Among the entire NSCLC cohort, highly statistically significant differences in survival were observed between surgically treated and non-surgically treated patients (*p* < 0.001). As shown in Figure 3, patients who underwent surgical resection demonstrated a markedly higher survival probability compared to patients who did not undergo surgery. The greatest decline in survival was observed between 12 and 24 months after diagnosis.

### 3.4. Analyses of Survival Probability in Patients Diagnosed with Small-Cell Lung Cancer

In SCLC patients treated with platinum-based regimens and etoposide, survival duration differed significantly by disease stage (*p* < 0.001). Survival outcomes worsened progressively with advancing clinical stage, being highest in stages I-II and dropping to their lowest in stage IV. For SCLC patients treated with platinum-based regimens and etoposide without radiotherapy, survival did not differ significantly by disease stage (*p* = 0.159). Early-stage disease (stages I–II) showed a slightly better prognosis; however, the lack of statistically significant differences precludes a definitive interpretation.

Among SCLC patients treated with platinum-based regimens combined with etoposide and radiotherapy, statistical analysis demonstrated significant differences in survival duration according to disease stage (*p* < 0.001). As expected, survival probability systematically declined, with the longest survival observed in stages I-II and the shortest in stage IV, despite the use of radiotherapy.

For SCLC patients receiving a single cycle of platinum-based chemotherapy combined with etoposide and radiotherapy, survival duration differed significantly according to disease stage (*p* = 0.0002). The longest survival was observed in patients with stage I–II disease, whereas the shortest survival was noted in those with stage IV disease. With increasing disease stage, a clear decline in survival probability was observed. Notably, the use of radiotherapy in combination with chemotherapy was associated with significantly improved survival outcomes compared to patients who received chemotherapy alone. Early-stage disease was characterized by a markedly more favorable prognosis, underscoring the importance of early diagnosis and the timely initiation of appropriate treatment.

Among SCLC patients who received two treatment cycles comprising platinum-based chemotherapy combined with etoposide and radiotherapy, statistical analysis did not reveal significant differences in survival duration according to disease stage. Despite the absence of statistical significance, the most favorable survival outcomes were observed in patients diagnosed at stages I–II. Over time, a gradual decline in survival probability was observed across all analyzed groups, with the largest decrease occurring between 12 and 36 months.

Among the study participants with small-cell lung cancer whose first of three or more treatment cycles consisted of platinum-based chemotherapy in combination with etoposide and radiotherapy, statistical analysis did not reveal significant differences in survival according to disease stage (*p* = 0.636). However, it can be noted that patients diagnosed with stage III disease and treated with this regimen exhibited a higher survival rate compared with patients who received only one or two cycles of the same treatment. It should also be noted that, over time, a gradual decline in survival probability was observed across all analyzed groups, particularly between 12 and 24 months.

**Figure 4:**
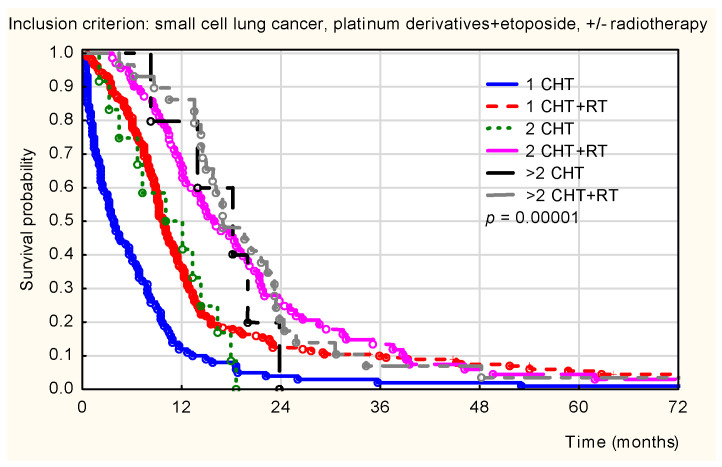
Kaplan–Meier overall survival curves for patients with small-cell lung cancer (SCLC), stratified by the number and modality of treatment cycles (chemotherapy [CHT] vs. chemoradiotherapy [CHT + RT]). Legend: 1 CHT (blue solid line), 1 CHT + RT (red dashed line), 2 CHT (green dotted line), 2 CHT + RT (pink solid line), >2 CHT (black solid line), and >2 CHT + RT (gray dashed line). Statistical significance: *p* < 0.001. Abbreviations: CHT, chemotherapy; RT, radiotherapy; PL + VEP, platinum derivatives and etoposide.

Statistically significant differences in survival probability were observed according to the treatment modality (*p* < 0.001). Figure 4 illustrates that the highest survival probability was achieved by patients treated with both chemotherapy and radiotherapy, regardless of the number of cycles, compared with patients who received chemotherapy alone.

To evaluate the impact of the investigated prognostic factors (clinical stage, surgical resection status, number of administered treatment cycles, and first-line treatment regimen) on overall survival in patients with small-cell lung cancer, a Cox proportional hazards regression analysis was performed. Initially, a univariable analysis was conducted to assess the effect of individual factors (Table 6), followed by a multivariable analysis to evaluate their simultaneous impact (Table 7). This two-step approach allowed for the precise determination of the independent influence of these factors on patient survival.

The data presented in the table indicate the following:

- The mortality risk for patients with small-cell lung cancer was highly dependent on clinical stage. For patients with stage IIIA disease, the risk was 69% higher, and for stage IIIB, it was 82% higher, compared to the reference group (stages I–II). Patients with stage IV disease exhibited the highest mortality risk, demonstrating a nearly three-fold increase compared to the reference group (HR = 2.81, 95% CI: 2.05–3.86, *p* < 0.001).
- Mortality risk was also significantly influenced by surgical resection status. Surgically treated patients had a 65% lower mortality risk compared to those who did not undergo surgery.
- For 96% of the small-cell lung cancer cohort, the first treatment cycle consisted of platinum-based chemotherapy combined with etoposide. The mortality risk for patients treated with etoposide monotherapy was nearly 4.5 times higher; however, this is primarily because etoposide monotherapy was reserved for patients ineligible for platinum-based combination regimens.
- Mortality risk was further impacted by the treatment modality and the number of cycles administered. Patients who received chemotherapy alone in the first cycle faced a higher mortality risk than those who received chemoradiotherapy. Moreover, an increased number of chemoradiotherapy cycles correlated with a lower mortality risk. In summary, based on the survival curves and univariable analysis, early clinical stage and the feasibility of surgical lung resection conferred a significant survival benefit for patients with small-cell lung cancer.

Multivariable analysis confirmed the highly significant impact of clinical stage and the superiority of chemoradiotherapy over chemotherapy alone regarding overall survival among patients with small-cell lung cancer. It also demonstrated that unfavorable prognostic factors include stages IIIB and IV disease, treatment with chemotherapy alone, and the administration of etoposide as monotherapy.

### 3.5. Analyses of Survival Probability in Patients Diagnosed with Non-Small-Cell Lung Cancer

#### 3.5.1. Analyses of Survival Probability in Patients Undergoing Surgical Treatment

Among surgically treated NSCLC patients, statistically significant differences in survival were observed according to disease stage (*p* = 0.0004). The highest survival probability was observed in patients diagnosed at stages I or II who underwent partial or complete lung resection. The shortest survival was noted in patients with stage IIIB disease, which is typically inoperable. For stages IIIA (operable) and IV, survival probabilities beyond 36 months remained relatively stable. These findings confirm the importance of effective treatment through surgical resection in eligible cases.

For patients receiving adjuvant chemotherapy, survival also differed significantly by disease stage (*p* = 0.002). The longest survival was observed in patients diagnosed at stage I, whereas the shortest survival was noted in those with stage IIIB disease. Over time, a gradual decline in survival probability was observed across all analyzed groups, with the most substantial decrease occurring in stages IIIA, IIIB, and IV, primarily between 12 and 36 months.

Overall, among surgically treated NSCLC patients, no statistically significant differences in survival probability were observed according to the general treatment regimen administered in the first cycle (*p* = 0.099). However, a gradual decline in survival probability was consistently observed over time across all groups.

For patients who underwent surgery and received a first cycle of platinum-based chemotherapy combined with vinorelbine, statistically significant differences in survival probability were observed according to disease stage (*p* = 0.034). Patients in stages I and II exhibited higher survival probabilities compared to those classified as stages IIIA, IIIB, and IV. Over time, a gradual decline in survival probability was observed in both groups.

Similarly, for surgically treated patients who received a first cycle of platinum-based chemotherapy combined with gemcitabine, statistically significant differences in survival were noted according to disease stage (*p* = 0.002). The longest survival was observed in patients diagnosed at stages I or II, with the smallest decline in survival probability occurring between 60 and 72 months after diagnosis. In contrast, patients with stages IIIA, IIIB, and IV disease experienced the sharpest decline in survival probability between 12 and 36 months following diagnosis and treatment.

When comparing specific adjuvant treatment regimens (platinum-based chemotherapy combined with either vinorelbine or gemcitabine), statistically significant differences in survival probability were observed (*p* = 0.041). A higher survival probability was noted in patients receiving adjuvant treatment with platinum derivatives and gemcitabine in the first cycle.

To assess the independent impact of prognostic factors (clinical stage, number of treatment cycles, and first-cycle treatment regimen) on overall survival in surgically treated NSCLC patients, a Cox proportional hazards regression analysis was performed. Initially, a univariable analysis evaluated the effect of individual factors (Table 8), revealing that only clinical stage was a significant predictor. The mortality risk for patients with stages IIIA, IIIB, and IV disease was 82% higher than for those with stages I and II.

#### 3.5.2. Analyses of Survival Probability in Patients Who Did Not Undergo Surgical Treatment

Among the NSCLC subgroup that did not undergo surgical treatment, statistically significant differences in survival probability were observed according to disease stage (*p* < 0.001). As expected, survival probabilities were highest in patients with early-stage disease. A gradual decline in survival probability was observed over time across all analyzed groups.

**Figure 5:**
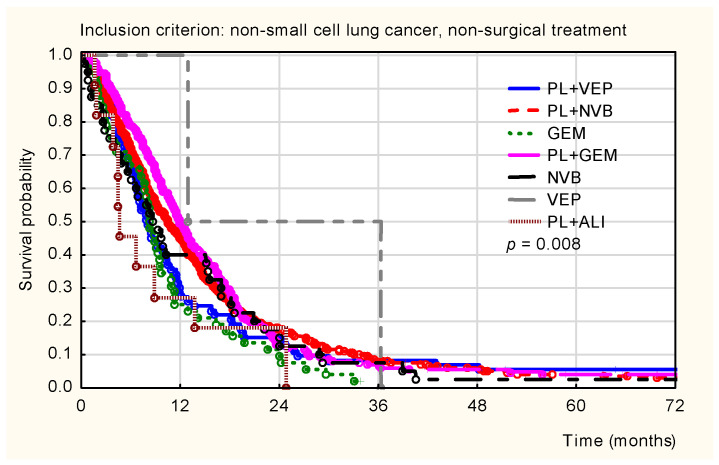
Kaplan–Meier overall survival curves for non-surgically treated patients with non-small-cell lung cancer (NSCLC), stratified by the first-line treatment regimen. The plot includes various regimens: platinum derivatives combined with etoposide (PL + VEP), vinorelbine (PL + NVB), gemcitabine (PL + GEM), or Pemetrexed (PL + ALI), as well as monotherapies with gemcitabine (GEM), vinorelbine (NVB), or etoposide (VEP). Statistical significance: *p* = 0.008.

In this non-surgical cohort, overall survival also differed significantly based on the first-cycle treatment regimen (*p* = 0.008; Figure 5). The highest survival probabilities were observed in patients treated with gemcitabine monotherapy or platinum-gemcitabine combination regimens. Across all treatment groups, the most pronounced decline in survival occurred during the first 24 months of follow-up.

Among non-surgical NSCLC patients who received a first cycle of platinum-based chemotherapy combined with vinorelbine, statistically significant differences in survival were observed according to disease stage (*p* = 0.0001). Survival probability was highest in patients with stage I, II, or IIIA disease, and lowest in those with stages IIIB and IV.

Data analysis also revealed statistically significant differences in survival based on the addition of radiotherapy in this specific subgroup (patients receiving first-line platinum agents and vinorelbine). Specifically, survival outcomes were significantly different when comparing patients who received chemotherapy alone versus those who received chemoradiotherapy. Furthermore, survival differed based on the total number of treatment cycles administered.

Similarly, among non-surgically treated NSCLC patients receiving a first cycle of platinum-based chemotherapy combined with gemcitabine, survival differed significantly according to disease stage (*p* = 0.044). Patients diagnosed at stages I and II exhibited the highest survival probabilities following this specific treatment regimen.

**Figure 6:**
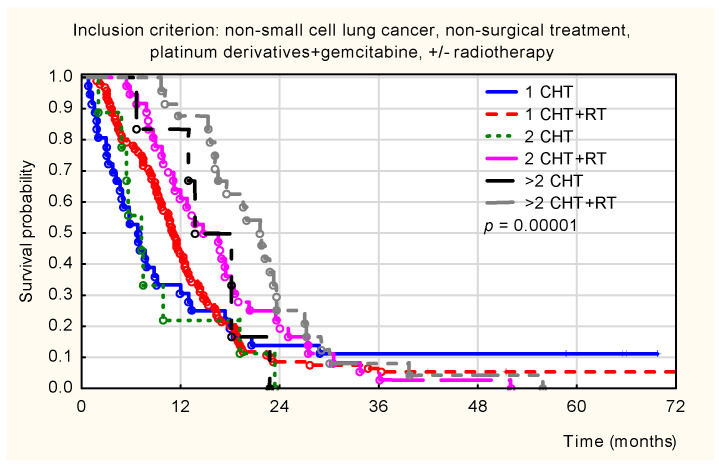
Kaplan–Meier overall survival curves for non-surgically treated patients with non-small-cell lung cancer (NSCLC) receiving the platinum derivative and gemcitabine (PL + GEM) regimen, stratified by the number of treatment cycles and the use of radiotherapy. Legend: 1 CHT cycle (blue solid line), 1 CHT + RT cycle (red dashed line), 2 CHT cycles (green dotted line), 2 CHT + RT cycles (pink solid line), >2 CHT cycles (black solid line), and >2 CHT + RT cycles (gray dashed line). Significance: *p* < 0.001.

Further analysis of this gemcitabine-treated cohort revealed a significant survival benefit associated with the addition of radiotherapy. As shown in Figure 6, patients who received chemoradiotherapy exhibited significantly higher survival probabilities compared to those treated with chemotherapy alone (*p* < 0.001). These survival differences were evident regardless of the total number of cycles administered.

Among non-surgically treated NSCLC patients who received a first cycle of platinum-based chemotherapy combined with etoposide, statistically significant differences in survival probability were observed according to clinical stage (*p* = 0.012). The highest survival probability was observed in patients diagnosed at stages I and II; from approximately 12 months onward, survival probabilities in this group remained relatively stable. The lowest survival probability was noted in patients with stage IIIB disease, reaching its minimum between 12 and 24 months after diagnosis.

Within this subgroup (platinum plus etoposide), statistically significant differences in survival were also observed based on the number of treatment cycles administered and the addition of radiotherapy. Patients receiving chemoradiotherapy exhibited higher survival probabilities compared to those treated with chemotherapy alone. The lowest survival probability was observed in patients receiving a single cycle of chemotherapy, whereas the highest was seen in those receiving more than two cycles of chemoradiotherapy.

For non-surgically treated NSCLC patients receiving first-line gemcitabine monotherapy, survival differed significantly by disease stage (*p* = 0.018), with the highest survival probability in stages I and II, and the lowest in stages IIIB and IV. Furthermore, survival in this gemcitabine cohort varied significantly based on the number of treatment cycles and the use of concurrent radiotherapy (*p* = 0.01). Chemoradiotherapy was associated with longer survival compared to chemotherapy alone. The lowest survival probability was noted in patients receiving exactly two cycles of chemotherapy alone, while the highest was observed in those treated with one or more cycles of chemoradiotherapy.

Conversely, among non-surgically treated NSCLC patients receiving first-line vinorelbine monotherapy, no statistically significant differences in survival probability were observed according to disease stage (*p* = 0.485). However, significant differences were noted based on the number of treatment cycles and the inclusion of radiotherapy (*p* = 0.002). The highest survival probability in this subgroup was observed in patients receiving two cycles of chemoradiotherapy, whereas the lowest was seen in those who received only a single cycle of chemotherapy.

To assess the independent impact of prognostic factors (clinical stage, number of treatment cycles, and first-cycle treatment regimen) on overall survival in non-surgically treated NSCLC patients, a Cox proportional hazards regression analysis was performed. Initially, a univariable analysis was conducted (Table 9), followed by a multivariable analysis (Table 10).

Based on the data presented in the table, the following conclusions were drawn regarding non-surgically treated NSCLC patients:

Mortality risk was significantly dependent on clinical stage. The analysis demonstrated a progressive increase in the mortality risk for patients with stage IIIA (HR = 1.40), stage IIIB (HR = 1.81), and stage IV (HR = 2.00) disease, compared to the reference group (stages I and II).

Regarding the first-line treatment regimen, 53% of patients received platinum-based chemotherapy combined with vinorelbine (the reference group). Only patients treated with gemcitabine in the first cycle exhibited a statistically significant increase in mortality risk. For all other treatment regimens, the mortality risk did not differ significantly from the reference group.

Mortality risk was also influenced by the number of treatment cycles and the treatment modality. The majority of patients (51.9%) received a single cycle of chemoradiotherapy, which served as the reference group. Patients who received only one cycle of chemotherapy had a 28% higher mortality risk compared to the reference group. While increasing the number of chemotherapy cycles yielded a non-significant reduction in mortality risk, increasing the number of chemoradiotherapy cycles resulted in a statistically significant decrease in mortality risk.

The multivariable analysis confirmed the findings obtained in the univariable analysis.

## 4. Discussion

Lung cancer is the most commonly diagnosed malignancy worldwide, ranking second in Poland and third in Europe. It constitutes the leading cause of cancer-related deaths in the Polish, European, and global populations [18]. Galli et al. [19] analyzed more than 12,500 patients with malignant lung tumors diagnosed in Switzerland between 1999 and 2015, reporting that 87% of men had non-small-cell lung cancer (NSCLC) and 13% had small-cell lung cancer (SCLC). Similar data were reported by Yang et al. at the Mayo Clinic in the United States [20]. In contrast, in the cohort analyzed in this study, the proportion of NSCLC was significantly lower (by approximately 15%), and SCLC significantly higher (by approximately 15%) than typically reported in the English-language literature. This discrepancy may be attributed to specific regional referral patterns during that decade. The Katowice Oncology Center was a highly specialized facility, meaning that patients with aggressive, rapidly progressing symptoms typical of SCLC—often heavy smokers from the heavily industrialized Silesian region—were more frequently and urgently directed here compared to general hospitals. When compared to other Polish studies, this difference was less pronounced. Słowik-Gabryelska and Sokołowski [21] reported in an epidemiological study conducted in Włocławek that 77% of patients had NSCLC and 23% had SCLC. Radzikowska and Głaz [22] analyzed male patients registered in Tuberculosis and Lung Disease Clinics in 1995, finding 80% with NSCLC and 20% with SCLC. Regarding treatment modalities, randomized trials comparing the efficacy of cisplatin and carboplatin (Skarlos et al. [23], Lassén et al. [24], Okamoto et al. [25], Rossi et al. [26]) in SCLC patients showed no significant differences in response rates or overall survival (OS). Similar conclusions were drawn for NSCLC patients [27]. A study comparing cisplatin versus carboplatin combined with etoposide (Klastersky et al. [28]) in advanced NSCLC patients showed a higher objective response rate for cisplatin (27% vs. 16%), whereas carboplatin was better tolerated and did not cause treatment-related deaths [29]. This favorable toxicity profile is a crucial consideration for maintaining treatment compliance in advanced-stage patients, such as the large cohort of stages IIIB and IV patients analyzed in our study. Similarly, Zatloukal et al. [30] evaluated OS in stage IIIB–IV NSCLC patients treated with cisplatin plus gemcitabine versus carboplatin plus gemcitabine and concluded that both regimens were comparable in efficacy and toxicity [31]. The proportion of patients diagnosed with localized lung cancer in the analyzed cohort was similar to that reported in the Polish National Cancer Registry [32]. However, regional-stage diagnoses were assigned to 46% of patients in the analyzed cohort versus 30% in the general Polish population, and metastatic disease to 35% versus 50% nationwide. These differences may be related to better access to specialized oncology care in the Silesian region. In 2017, a national strategy was recommended in Poland, focusing on the early detection of lung cancer and the implementation of advanced treatment methods. Key elements include primary prevention to reduce risk factors, promotion of healthy behaviors, improving the efficiency of early detection, enhancing diagnostic and treatment quality, and ensuring access to the latest research and therapies available internationally [33]. Low-dose computed tomography (LDCT) has emerged as an important tool for early diagnosis, potentially reducing lung cancer mortality by up to 20% [34]. In the present study, which included 1431 men with malignant lung tumors treated between 2002 and 2012, partial or total lung resection was performed in 13.1% of patients overall. Among NSCLC patients, 18% underwent surgery, while only 2.5% of SCLC patients underwent resection. These surgical resection rates are significantly lower than those reported in broader national registries from Poland in the 1990s or international studies from the United States [35]. Historical data show that among 4619 men with lung cancer registered in Tuberculosis and Lung Disease Clinics in 1995, 20.9% underwent surgical treatment [36]. At the Mayo Clinic, 37% of all analyzed patients underwent lung resection, and among men with NSCLC, 48% underwent surgery. The proportion of patients undergoing surgery inherently varies according to clinical stage. In the present cohort, surgical resection was performed in up to 50% of stage I patients and 43% of stage II patients, compared to 14% in stage IIIA, 2% in stage IIIB, and slightly over 2% in stage IV. At the Mayo Clinic, these respective surgical rates were 93%, 87%, 52%, 24%, and 11% [20].

In the analyzed cohort, the 5-year overall survival (OS) rate for SCLC patients was 3.7%, while for NSCLC patients, it was 8.1%. In comparison, Yang et al. [20] reported a 5-year OS of 9% for SCLC and 19% for NSCLC among a cohort of 5628 patients. One-year OS rates for NSCLC patients in stages IIIB and IV were comparable to those observed at the Medical University Chemotherapy Clinic in Łódź, where 33.65% of patients in stages IIIB–IV survived one year, versus 39.3% (stage IIIB) and 37.1% (stage IV) in the present cohort [36]. As expected, survival rates were significantly higher in patients with a lower clinical stage for both SCLC and NSCLC. For SCLC, the 5-year OS was 16.7% in stages I and II, dropping to 1.1% in stage IV. For NSCLC, these rates were 43.2% and 1.9%, respectively. Similar results were reported by the Mayo Clinic; the 5-year OS for limited-stage SCLC was 17% versus 2% for extensive-stage disease, and for NSCLC, it was 59% in stage IA versus 2% in stage IV [20].

Patients who underwent partial or total lung resection demonstrated markedly better survival outcomes. Among surgically treated NSCLC patients, the 5-year OS was 28.5%, compared to only 3.8% in patients who did not undergo surgery. The majority of patients who underwent surgery were in stages I and II, exhibiting a 5-year OS of 35.8%. Nevertheless, these outcomes are still lower than those reported by other authors. Chabowski et al. [37] reported a 5-year OS of 49.1% among 431 surgically treated NSCLC patients in Warsaw, while Goya et al. [38] reported 52.6% among 6644 patients in Japan.

Among SCLC patients, surgical resection was performed in only 2.5% of cases, all of whom had limited-stage disease. The 5-year OS for this subgroup was 15.3%, versus 3.4% among patients managed non-surgically. In a similar context, Rostad et al. [39] reported that partial or total lung resection was performed in 1.6% of SCLC patients in Norway over a 7-year period, with a 5-year OS of 44.6% in stages IA–IB for surgically treated patients versus 11.3% for those managed non-surgically. Similar benefits of surgical intervention in early-stage SCLC have been reported by Varlotto et al. [40], Combs et al. [41], and Yao et al. [42]. These findings suggest that surgical treatment should be more frequently considered and applied in patients with early-stage lung cancer. Conversely, among non-surgically treated NSCLC patients, eleven different first-line treatment regimens were administered. The 5-year OS rates in this group were exceptionally low, with a maximum of 5.5% observed for the regimen consisting of platinum derivatives combined with etoposide.

Table 11 summarizes the 1-year overall survival (OS) outcomes reported in clinical studies using the same treatment regimens as those administered to the patients in the present analysis. In our study, the highest 1-year OS was observed in patients across all stages treated with platinum derivatives combined with gemcitabine (50.5%). Comparable results for patients with advanced non-small-cell lung cancer (NSCLC) were reported by Jassem et al. [43]. In contrast, other authors listed in Table 11 reported 1-year survival rates for this regimen that were approximately 10 percentage points lower than our findings [43,44,45,46]. The regimen consisting of platinum derivatives combined with vinorelbine ranked second in terms of 1-year survival (45.2%). For patients with advanced NSCLC, the present analysis yielded results comparable to the existing literature (stage IIIB: 35%, stage IV: 38%) [47,48]. Conversely, the 1-year OS for advanced NSCLC patients treated with platinum derivatives plus etoposide was lower than previously reported [29,43,44,45,46] (stage IIIB: 10%, stage IV: 27%; literature range: 26–37%).

Among NSCLC patients who underwent partial or total lung resection, eight different adjuvant regimens were applied in the first cycle. The highest survival rates were observed in patients receiving platinum derivatives with gemcitabine or platinum derivatives with vinorelbine (5-year OS: 36.1% and 24.9%; 2-year OS: 71.1% and 52.5%, respectively), which were significantly higher than in the non-surgically treated group. Uramoto et al. [52] reported a 2-year OS of 79.1% in a phase II randomized trial of NSCLC patients receiving adjuvant carboplatin plus gemcitabine. Douillard et al. [53] evaluated adjuvant cisplatin plus vinorelbine, achieving a 5-year OS of 55.1%. Furthermore, a pooled analysis by the Lung Adjuvant Cisplatin Evaluation (LACE) Collaborative Group of 4584 patients showed that cisplatin plus vinorelbine provided slightly better outcomes than cisplatin combined with other agents, such as etoposide or vinblastine [54]. Multiple studies have demonstrated that cisplatin-based adjuvant chemotherapy significantly improves survival in NSCLC patients [55,56], a conclusion consistent with our data. In the studied cohort, neoadjuvant chemotherapy was applied in only 16 NSCLC patients, a sample size precluding the possibility of drawing statistically robust conclusions [57,58].

Regarding small-cell lung cancer (SCLC), platinum derivatives combined with etoposide were used in the first cycle in over 95.5% of cases. This regimen remains the primary recommendation by the Polish Society of Clinical Oncology for all clinical stages [59]. Notably, survival rates for SCLC patients receiving chemoradiotherapy were significantly higher than for those treated with chemotherapy alone. The two-year OS for patients treated with concurrent chemoradiotherapy versus chemotherapy alone was as follows: stages I and II (40.8% vs. 14.3%), stage IIIA (18.4% vs. 11.1%), stage IIIB (13.7% vs. 0%), and stage IV (6.9% vs. 1.5%). Sas-Korczyńska et al. [60] reported a 2-year OS of 39.3% in limited-stage SCLC patients receiving concurrent cisplatin plus etoposide chemoradiotherapy.

The present study covers the period 2002–2012, when first-line treatment for NSCLC in Poland was primarily limited to conventional chemotherapy. Over the past decade, numerous targeted molecular agents have been introduced, particularly for adenocarcinoma, to exploit specific molecular aberrations in tumor pathogenesis. For patients with EGFR mutations (10–20% of NSCLC [61]) or ALK rearrangements (approximately 5% of NSCLC [62,63]), tyrosine kinase inhibitors (TKIs) are now standard. Several of these are currently reimbursed in Poland, including EGFR TKIs (erlotinib, gefitinib, and afatinib) and ALK TKIs (crizotinib, alectinib, and ceritinib), as well as immunotherapy with pembrolizumab [64].

Atezolizumab was the first new drug in 40 years approved for the first-line treatment of advanced SCLC (EU—September 2019). In July 2020, durvalumab also received a positive opinion from the EMA for this indication. These agents represent the primary immunotherapy options currently approved for this setting, although they were not yet reimbursed in Poland as of April 2021 [65,66]. Their introduction has significantly improved the prognosis for patients with advanced lung cancer.

Reducing lung cancer mortality is inextricably linked to early diagnosis and subsequent curative-intent surgical resection. Earlier screening methods, such as chest X-rays and sputum cytology, did not yield the expected survival benefits [67,68]. However, since the 1990s, low-dose computed tomography (LDCT) has been extensively studied as a screening tool. Observational studies in the United States, Germany, and Japan demonstrated that LDCT can detect a high proportion of stage I lung cancers (approximately 85–93%). Following multicenter randomized trials, the United States implemented routine LDCT screening in 2015 for high-risk populations. In Europe, while LDCT is not yet universally implemented, it is strongly recommended by several professional societies, including the European Respiratory Society (ERS), the European Society of Radiology (ESR) [69], the European Society for Medical Oncology (ESMO) [70], and the European Society of Thoracic Surgeons (ESTS) [71].

In Poland, the “National Program for Early Detection of Lung Cancer (WWRP) using Low-Dose CT” has been conducted since 2018. This program combines primary and secondary prevention to improve lung cancer awareness among both the general public and healthcare personnel. Patients undergo LDCT as part of this initiative, and the long-term impact on survival will be fully evaluated following the program’s completion [72].

### 4.1. Study Strengths and Limitations

This study has several limitations that must be acknowledged. First, its retrospective, single-center design inherently limits the generalizability (external validity) of the findings to the broader, national population. Second, the reliance on historical, archival medical records from 2002 to 2012 introduces potential selection and information biases. For instance, clinical staging according to the TNM classification was not consistently recorded in all original charts and often had to be retrospectively determined based on available imaging and histopathological reports, which may affect internal validity. Third, the study timeframe predates the widespread introduction and reimbursement of modern targeted therapies (e.g., *EGFR* or *ALK* inhibitors) and immune checkpoint inhibitors in Poland. Consequently, the survival outcomes observed here reflect historical chemotherapy and radiochemotherapy protocols rather than contemporary precision oncology.

Despite these limitations, a major strength of this study is the large, homogeneous cohort of 1431 male patients evaluated over a long-term follow-up period. This comprehensive dataset provides a robust and valuable historical baseline for assessing the evolution of lung cancer care and the long-term impact of standard therapeutic modalities in Poland.

### 4.2. Comparative Analysis: Retrospective Outcomes vs. “Lung Cancer Mission 2024–2034” Strategic Goals

1. Long-Term Survival Forecasts and Mortality Goals

The significance of the retrospective data for 2002–2012 becomes particularly evident when contrasted with the measurable key performance indicators (KPIs) established by experts in the latest strategic document, “Lung Cancer Mission 2024–2034” in Poland. The primary objective of the strategy for the coming decade is to increase the national 5-year overall survival rate from the current level of approximately 14.4% to an absolute minimum of 20% by 2034. To achieve these forecasts, Polish oncology aims to double the number of patients undergoing curative-intent treatment compared to previous years. Consequently, our cohort represents an era in which radical cure was feasible only for a very narrow, highly selected group of patients [73].

2. Paradigm Shift in Early Detection and Resectability

The primary barrier resulting in the low percentage of men undergoing surgical resection in our study group was the lack of effective early diagnostic tools. According to projections for 2025–2034, the proportion of lung cancers diagnosed at early stages (stages I and II) is expected to increase from the current 20% to 30–40%. This shift will be facilitated by the implementation of the National Lung Cancer Early Detection Program using low-dose computed tomography (LDCT). Data from pilot editions of the program indicate that the widespread implementation of LDCT screening in high-risk populations can reduce lung cancer-specific mortality by up to 20% to 26% [74].

3. Widespread Adoption of Advanced Molecular Diagnostics (Next-Generation Sequencing)

The 2002–2012 period analyzed in this study represents an era of empirical selection of cytotoxic chemotherapy. Currently, an initiative is being implemented within Polish oncology to ensure that 100% of patients diagnosed with advanced non-squamous lung cancer (adenocarcinoma) are guaranteed multigene molecular profiling via next-generation sequencing (NGS) prior to making first-line treatment decisions. In 2024 alone, over 10,500 such tests were performed in Poland. The implementation of personalized therapies targeting specific molecular alterations (such as EGFR, ALK, ROS1, or KRAS G12C) is expected to fundamentally alter patient survival trajectories compared to our retrospective data [75,76].

### 4.3. Evolution of Treatment Modalities: From Cytotoxicity to Precision Medicine

Analyzing data from the last 10 years, the progress in lung cancer treatment has been tremendous, with therapeutic efficacy increasing significantly compared to the 2002–2012 period. Surgical treatment remains the absolute cornerstone of curative-intent modalities; however, despite the excision of altered tissues, there is still a high risk of micrometastases and disease recurrence. Therefore, the primary therapeutic goal in recent years has been the broad implementation of neoadjuvant (preoperative) and adjuvant (postoperative) methods based on immunotherapy, molecularly targeted therapy, and chemotherapy. Given that this malignancy is frequently diagnosed at locally advanced or metastatic stages, radical surgical interventions can currently be applied in only approximately 20% of patients [77].

In patients with locally advanced non-small-cell lung cancer (NSCLC) and small-cell lung cancer (SCLC), radiotherapy plays a pivotal role, often administered in combination with chemotherapy (concurrent or sequential chemoradiotherapy). It constitutes the gold standard for the radical treatment of patients with clinical stage III disease who are inoperable, as well as those with severe medical contraindications to surgery. Recent years in radiotherapy have been a period of immense innovation—advanced equipment allows for the generation of radiation with precisely tailored volumes and doses. Image-guided radiation therapy (IGRT) and stereotactic body radiotherapy (SBRT) are widely utilized. Modern radiotherapy relies on the integration of imaging studies (computed tomography, magnetic resonance imaging, or PET-CT), which enables a highly individualized approach to each patient and the maximally precise destruction of tumor cells while sparing healthy tissues [78,79].

Currently, Poland operates a highly comprehensive national drug program (B.6) for patients with non-small-cell lung cancer. Approved molecularly targeted therapies cover patients with identified pathogenic variants in the EGFR, KRAS (G12C), BRAF, and MET genes, as well as those with ALK, ROS1, and NTRK1/2/3 gene rearrangements. Within these indications, treatments utilized include EGFR tyrosine kinase inhibitors (e.g., osimertinib, afatinib, erlotinib, and gefitinib), ALK/ROS1/NTRK-targeted agents (crizotinib, alectinib, lorlatinib, and entrectinib), and KRAS G12C inhibitors (sotorasib). Furthermore, immunotherapy in patients with NSCLC is administered across the following diverse regimens:

- In patients with stage IIIA and IIIB disease, following concurrent chemoradiotherapy, consolidation therapy with durvalumab is the standard of care;
- In the first-line treatment of patients with PD-L1 expression in at least 50% of tumor cells and the absence of actionable pathogenic variants (e.g., EGFR/ALK), pembrolizumab, cemiplimab, or atezolizumab is implemented;
- Following the failure of prior treatment modalities (chemotherapy), immunotherapy (e.g., nivolumab or atezolizumab) can be used in both squamous and non-squamous (adenocarcinoma) histological subtypes;
- The program also encompasses NSCLC patients with the KRAS G12C mutation following the failure of previous lines of treatment [80].

### 4.4. Recent Breakthroughs and Regulatory Approvals in Lung Cancer Therapy

A tremendous breakthrough has also occurred in the treatment of small-cell lung cancer (SCLC). For decades, the foundation of SCLC treatment, regardless of the stage, was exclusively chemotherapy based on platinum derivatives and etoposide. Currently, the new first-line standard of care for patients with extensive-stage disease is chemoimmunotherapy—a combination of traditional chemotherapy with atezolizumab or durvalumab. Furthermore, dual immunotherapy (nivolumab in combination with ipilimumab) is successfully utilized within the first-line treatment program for pleural mesothelioma. This progress is incredibly dynamic. By 1 October 2024 alone, the following key reimbursement and regulatory decisions were made:

1. Poland (AOTMiT): The Transparency Council issued a positive statement regarding the reimbursement of tremelimumab in combination with durvalumab.
2. European Medicines Agency (EMA): The EMA registered and expanded the indications for: serplulimab in the treatment of SCLC patients; sugemalimab in combination with platinum-based chemotherapy in NSCLC without pathogenic variants in the *EGFR*, *ALK*, *ROS1*, or *RET* genes; encorafenib in combination with binimetinib for patients with *BRAF* V600E-mutated NSCLC; amivantamab in a regimen with carboplatin and pemetrexed for NSCLC patients with pathogenic variants in the *EGFR* gene (ex19 or ex21); atezolizumab as a first-line monotherapy for patients with advanced NSCLC who are ineligible for platinum-based therapy; tislelizumab in combination with pemetrexed and chemotherapy for the first-line treatment of *EGFR*/*ALK*-negative, non-squamous NSCLC with PD-L1 expression ≥ 50%; and osimertinib in combination with chemotherapy for the first-line treatment of advanced NSCLC patients with an *EGFR* mutation.
3. US Food and Drug Administration (FDA): The FDA approvals included pembrolizumab in combination with chemotherapy for the first-line treatment of unresectable advanced pleural mesothelioma; amivantamab in combination with chemotherapy for NSCLC patients with *EGFR* pathogenic variants experiencing progression during or after tyrosine kinase inhibitor therapy; osimertinib for the treatment of locally advanced, unresectable (stage III) NSCLC following concurrent or sequential chemoradiotherapy (*EGFR* ex19/ex21 L858R mutations); nivolumab in combination with chemotherapy for neoadjuvant treatment followed by adjuvant monotherapy in patients with resectable NSCLC (tumor ≥ 4 cm and/or node-positive); durvalumab in neoadjuvant treatment combined with chemotherapy, followed by adjuvant durvalumab monotherapy post surgery; lazertinib in combination with amivantamab for the first-line treatment of patients with *EGFR* mutations; and atezolizumab combined with hyaluronidase in an innovative subcutaneous injection formulation [81].

### 4.5. Epidemiological Landscape and Challenges in Poland

Lung cancer remains one of the greatest challenges of contemporary Polish oncology, standing as the absolute leader in terms of mortality. It accounts for nearly 24% of all cancer-related deaths in the country, which translates to approximately 23,000 deaths annually. However, unprecedented changes are currently occurring within the epidemiological structure. Since the beginning of the 21st century, a systematic decline in the incidence and standardized mortality rates among men (by approximately 22%) has been observed, which is a direct result of long-term anti-tobacco campaigns and industrial transformation. This phenomenon sharply contrasts with the dramatic, nearly 95% increase in incidence among the Polish female population, for whom lung cancer has now become a more frequent cause of death than breast cancer.

Regardless of gender, delayed diagnosis remains a fundamental problem—up to 80% of patients in Poland are diagnosed at locally advanced or metastatic stages (stages III and IV), a point at which the possibilities for applying radical surgical treatment are drastically limited. This results in a 5-year survival rate ranging from 14.5% to 17.8%, which continues to place Poland below the average of Western European and Scandinavian countries, where these values exceed 20% [82].

## 5. Conclusions and Recommendations

Based on the findings of this 10-year retrospective cohort study and their confrontation with contemporary oncological standards, we draw the following conclusions:

Clinical Practice: Advanced disease stage at the initiation of treatment significantly shortens overall survival, regardless of the therapy modality employed. Survival is markedly prolonged in patients undergoing surgical resection; therefore, clinical efforts must prioritize early intervention and maximize surgical eligibility. In non-surgical cases, multimodal treatments (concurrent or sequential chemoradiotherapy) must remain the gold standard, as they yield superior survival compared to chemotherapy alone. Furthermore, while our data identified platinum–gemcitabine as a superior historical regimen, modern clinical practice should now mandate the integration of immunotherapy and molecularly targeted agents as the new standard of care to surpass these historical survival baselines.

Health Policy: The overwhelming impact of early diagnosis on survival underscores the critical need for healthcare policymakers to expand and fully fund national screening programs utilizing low-dose computed tomography (LDCT). The strategic goal of the “Lung Cancer Mission 2024–2034” to increase the 5-year survival rate in Poland to a minimum of 20% can only be achieved by doubling the number of patients treated with curative intent. To this end, systemic barriers to advanced molecular diagnostics, specifically next-generation sequencing (NGS), must be removed to ensure 100% of eligible patients have access to personalized first-line therapies.

Education and Prevention: There is a pressing need for educational initiatives targeting high-risk populations, particularly in industrialized regions like Silesia, to promote smoking cessation and symptom awareness. Given the dramatic 95% increase in lung cancer incidence among Polish women, gender-specific prevention programs are urgently required. Equally important is the continuous education of primary care physicians to accelerate diagnostic pathways.

Future Research: Future prospective, multi-center studies are required to build upon these findings. Our 10-year data establish a crucial historical baseline for the “pre-precision medicine” era in Poland. Future research should rigorously compare these results with contemporary cohorts treated under the current B.6 drug program to quantify the real-world survival benefit of immunotherapy and TKIs in the Polish population.

## Figures and Tables

**Table 1:** Overview of actionable gene alterations in non-small-cell lung cancer (NSCLC) and their clinical characteristics.

Gene	Clinical and Demographic Characteristics	Associated Treatment
EGFR	Lung adenocarcinoma; more frequent in never-smokers, women, and individuals of Asian descent [6].	Treated with EGFR tyrosine kinase inhibitors (EGFR-TKIs). Exon 20 insertions are associated with TKI resistance [6].
ALK	Predominantly found in never-smokers; associated with an increased risk of venous thromboembolism [7].	Treated with ALK inhibitors [7].
ROS1	Associated with an increased incidence of venous thromboembolic events [8].	Targeted therapy available [8].
BRAF (V600E)	Found in older patients regardless of smoking history; slightly more frequent in women. No distinct clinical phenotype [9].	Targeted therapy available. V600E accounts for ~50% of BRAF mutations [9].
MET (ex14 skipping)	Observed in older patients regardless of smoking history; slightly more common in women. Found in 20–30% of pulmonary sarcomatoid carcinomas [10].	Targeted therapy available [10].
RET	More frequently identified in never-smokers with lung adenocarcinoma [11].	First-line targeted therapy approved [11].
KRAS	More commonly detected in smokers compared with other actionable alterations [12]	Standard care includes first-line immunotherapy with or without chemotherapy [12].
ERBB2	More frequent in women, younger patients, never-smokers, and individuals of Asian ethnicity [13].	HER2 mutation must be distinguished from HER2 overexpression (assessed by IHC) [13].
NTRK	Rare; more commonly reported in younger patients, never-smokers, and adenocarcinoma cases [14].	Treated with TRK inhibitors. If unavailable, standard immunotherapy/chemotherapy is considered [14].

**Table 2:** Distribution of lung cancer histological types across age groups.

Age Group		<45	45–49	50–54	55–59	60–64	65–69	70–74	75+	Total	
Histological Type											
SCLC		10	29	61	75	94	66	66	29	430	
NSCLC	Squamous-Cell Carcinoma	15	30	71	111	128	133	86	38	612	1001
	Adenocarcinoma	10	11	30	31	47	40	22	5	196	
	Others	6	13	30	38	37	31	27	11	193	
Total		41	83	192	255	306	270	201	83	1431	
Overall		2.9%	5.8%	13.4%	17.8%	21.4%	18.9%	14.0%	5.8%	100%	

Abbreviations: NSCLC—non-small-cell lung cancer; SCLC—small-cell lung cancer.

**Table 3:** Distribution of disease stage according to the TNM classification at diagnosis by lung cancer histological type.

Histological Type Clinical Stage	SCLC	NSCLC			Total
		Squamous-Cell Carcinoma	Adenocarcinoma	**Others**	
I A	6	8	7	2	23 (1.6%)
I B	6	15	4	0	25 (1.7%)
II A	21	51	23	8	103 (7.2%)
II B	24	59	19	13	115 (8.0%)
III A	80	187	41	55	363 (25.4%)
III B	113	112	27	44	296 (20.7%)
IV	180	180	75	71	506 (35.4%)
Total	430	612	196	193	1431

Abbreviations: NSCLC—non-small-cell lung cancer; SCLC—small-cell lung cancer; stages I–IV were defined according to the TNM classification (8th edition).

**Table 4:** Distribution of the number of treatment cycles, including chemotherapy and radiotherapy.

	Cycle RT	Without RT	1 Cycle RT	2 Cycles RT	3 Cycles RT	4 Cycles RT	5 Cycles RT	Total
Cycle CHT								
1		346	406	209	70	9	1	1041 (72.7%)
2		49	114	75	25	5	1	269 (18.8%)
3		16	27	36	12	2	0	93 (6.5%)
4		2	12	4	6	0	0	24 (1.7%)
5		0	2	2	0	0	0	4 (0.3%)
Total		413 (28.9%)	561 (39.2%)	326 (22.8%)	113 (7.9%)	16 (1.1%)	2 (0.1%)	1431 (100%)

Abbreviations: CHT—chemotherapy; RT—radiotherapy.

**Table 5:** Distribution of first-cycle treatment regimens according to lung cancer histological type.

Treatment Regimen in the First Cycle	Non-Small-Cell Lung Cancer	Small-Cell Lung Cancer
	Number of Cases	
PL + VEP	82	411
PL + GEM	262	
PL + NVB	525	5
GEM	60	
NVB	47	
VEP	2	12
PL + ALI	17	
ADR + E + VCR	1	2
PL + VBL	1	
PL + ADR	1	
PL + DOC	1	
ADR	1	
E + VCR	1	
Total	1001	430

Abbreviations: PL—platinum derivatives; VEP—etoposide; GEM—gemcitabine; NVB—vinorelbine; ALI—pemetrexed; VBL—vinblastine; ADR—doxorubicin; E—etoposide; VCR—vincristine; DOC—docetaxel.

**Table 6:** Univariable analysis of prognostic factors affecting survival probability in patients with small-cell lung cancer.

Prognostic Factors		No. of Cases (*n*)	Hazard Ratio (HR)	95% CI for HR	*p*-Value
Clinical Stage	I + II	57	1.0		
	III A	80	1.69	1.18–2.40	**0.004**
	III B	113	1.82	1.30–2.54	**0.0005**
	IV	180	2.81	2.05–3.86	**0.0001**
Surgical Resection (Partial or Total)	Non-operated	419	1.0		
	Operated	11	0.35	0.17–0.71	**0.004**
First-line Treatment Regimen	Platinum derivatives + VEP	411	1.0		
	VEP	12	4.48	2.49–8.04	**0.0001**
	Platinum derivatives + NVB; ADR + E + VCR	7	0.81	0.36–1.82	0.610
Number of Treatment Cycles	1 cycle CHT + RT	201	1.0		
	1 cycle CHT	110	2.18	1.72–2.77	**0.0001**
	2 cycles CHT	13	1.21	0.69–2.12	0.512
	>2 cycles CHT	5	0.72	0.30–1.75	0.469
	2 cycles CHT +RT	72	0.67	0.51–0.89	**0.005**
	>2 cycles CHT + RT	29	0.61	0.41–0.90	**0.014**

Bolded rows indicate statistically significant prognostic factors (*p* < 0.05) identified in the multi-variable Cox proportional hazards regression model. Abbreviations: HR, hazard ratio; 95% CI, 95% confidence interval; I–IV—stages were defined according to the TNM classification (8th edition), CHT—chemotherapy, RT—radiotherapy, VEP—etoposide, NVB—vinorelbine, ADR—doxorubicin, E—etoposide, VCR—vincristine.

**Table 7:** Multivariable analysis of prognostic factors affecting survival probability in patients with small-cell lung cancer.

Prognostic Factors		No. of Cases (*n*)	Hazard Ratio (HR)	95% CI for HR	*p*-Value
Clinical Stage	I + II	57	1.0		
	III A	80	1.31	0.91–1.91	0.149
	III B	113	1.61	1.13–2.30	**0.008**
	IV	180	2.16	1.54–3.04	**0.0001**
First-line Treatment Regimen	Platinum derivatives + VEP	411	1.0		
	VEP	12	3.79	2.09–6.87	**0.0001**
	Platinum derivatives + NVB; ADR + E + VCR	7	0.70	0.30–1.61	0.402
Number of Treatment Cycles	1 cycle CHT + RT	201	1.0		
	1 cycle CHT	110	2.05	1.60–2.62	**0.0001**
	≥2 cycles CHT	18	0.96	0.59–1.58	0.878
	2 cycles CHT + RT	72	0.74	0.56–0.98	**0.035**
	≥3 cycles CHT + RT	29	0.58	0.39–0.86	**0.007**

Bolded rows indicate statistically significant prognostic factors (*p* < 0.05) identified in the multi-variable Cox proportional hazards regression model. Abbreviations: HR, hazard ratio; 95% CI, 95% confidence interval; I–IV—stages were defined according to the TNM classification (8th edition), CHT—chemotherapy, RT—radiotherapy, VEP—etoposide, NVB—vinorelbine, ADR—doxorubicin, E—etoposide, VCR—vincristine.

**Table 8:** Univariate analysis of the impact of prognostic factors on survival probability in patients with non-small-cell lung cancer who underwent surgical treatment.

Prognostic Factors		No. of Cases (*n*)	Hazard Ratio (HR)	95% CI for HR	*p*-Value
Clinical Stage	I + II	108	1.0		
	III A + III B + IV	69	1.82	1.28–2.57	**0.0008**
First-line Treatment Regimen	Platinum derivatives + NVB	90	1.0		
	Platinum derivatives + GEM	55	0.72	0.48–1.08	0.109
	Others	32	1.02	0.65–1.58	0.945
Number of Treatment Cycles	1 cycle CHT	201	1.0		
	≥2 cycles CHT	110	1.48	0.72–3.04	0.281
	1 cycle CHT + RT	18	1.57	1.04–2.38	0.031

Bolded rows indicate statistically significant prognostic factors (*p* < 0.05) identified in the multi-variable Cox proportional hazards regression model. Abbreviations: HR, hazard ratio; 95% CI, 95% confidence interval; I–IV—stages were defined according to the TNM classification (8th edition), CHT—chemotherapy, RT—radiotherapy, NVB—vinorelbine, GEM—gemcitabine.

**Table 9:** Univariate analysis of the impact of prognostic factors on survival probability in patients with non-small-cell lung cancer who did not undergo surgical treatment.

Prognostic Factors		No. of Cases (*n*)	Hazard Ratio (HR)	95% CI for HR	*p*-Value
Clinical Stage	I + II	101	1.0		
	III A	232	1.40	1.09–1.78	**0.008**
	III B	177	1.81	1.41–2.34	**0.0001**
	IV	314	2.00	1.58–2.53	**0.0001**
First-line Treatment Regimen	Platinum derivatives + NVB	435	1.0		
	Platinum derivatives + VEP	73	1.16	0.90–1.49	0.251
	GEM	52	1.41	1.06–1.89	**0.020**
	Platinum derivatives + GEM	207	0.95	0.80–1.12	0.546
	NVB	40	1.10	0.79–1.52	0.586
	Others	17	1.48	0.90–2.45	0.122
Number of Treatment Cycles	1 cycle CHT + RT	428	1.0		
	1 cycle CHT	183	1.28	1.07–1.53	**0.008**
	2 cycles CHT	27	0.88	0.59–1.30	0.517
	>2 cycles CHT	10	0.80	0.43–1.49	0.477
	2 cycles CHT + RT	118	0.65	0.53–0.80	**0.0001**
	>2 cycles CHT + RT	58	0.51	0.39–0.68	**0.0001**

Bolded rows indicate statistically significant prognostic factors (*p* < 0.05) identified in the multi-variable Cox proportional hazards regression model. Abbreviations: HR, hazard ratio; 95% CI, 95% confidence interval; I–IV—stages were defined according to the TNM classification (8th edition), CHT—chemotherapy, RT—radiotherapy, VEP—etoposide, NVB—vinorelbine, GEM—gemcitabine.

**Table 10:** Multivariable analysis of prognostic factors affecting survival probability in patients with non-small-cell lung cancer who did not undergo partial or complete lung resection.

Prognostic Factors		No. of Case (*n*)	Hazard Ratio (HR)	95% CI for HR	*p*-Value
Clinical Stage	I + II	101	1.0		
	III A	232	1.40	1.09–1.79	**0.007**
	III B	177	1.94	1.50–2.52	**0.0001**
	IV	314	2.04	1.61–2.59	**0.0001**
First-line Treatment Regimen	Platinum derivatives + NVB	435	1.0		
	Platinum derivatives + VEP	73	1.16	0.90–1.50	0.243
	GEM	52	1.56	1.16–2.09	**0.003**
	Platinum derivatives + GEM	207	0.96	0.81–1.14	0.639
	NVB	40	1.00	0.72–1.39	0.995
	Others	17	1.25	0.76–2.07	0.385
Number of Treatment Cycles	1 cycle CHT + RT	428	1.0		
	1 cycle CHT	183	1.24	1.03–1.49	**0.023**
	>2 cycles CHT	37	0.79	0.56–1.11	0.167
	2 cycles CHT + RT	118	0.67	0.54–0.82	**0.0001**
	>2 cycles CHT + RT	58	0.48	0.36–0.64	**0.0001**

Bolded row indicate statistically significant prognostic factors (*p* < 0.05) identified in the multivariable Cox proportional hazards regression model. Abbreviations: HR, hazard ratio; 95% CI, 95% confidence interval; I–IV—stages were defined according to the TNM classification (8th edition), CHT—chemotherapy, RT—radiotherapy, VEP—etoposide, NVB—vinorelbine, GEM—gemcitabine.

**Table 11:** Comparison of survival in patients with advanced non-small-cell lung cancer (NSCLC) according to the first-line chemotherapy regimen.

Diagram	Author	1-Year Survival (%)
PL + NVB	Wozniak et al. [49]	40%
	Kelly et al. [50]	36%
PL + GEM	Liu et al. [51]	32%
	Schiller et al. [47]	36%
	Hoang et al. [48]	36%
	Smit. et al. [44]	33%
	Jassem et al. [43]	45%
PL + VEP	Liang et al. [45]	32%
	Steuer et al. [46]	37%
	Liu et al. [51]	26%
	Klastersky et al. [28]	34%
	Hoang et al. [48]	32%
NVB	Wozniak et al. [49]	35%

Abbreviations: PL + NVB—platinum derivatives and vinorelbine, PL + GEM—platinum derivatives and gemcitabine, PL + VEP—platinum derivatives and etoposide, NVB—vinorelbine.

## Data Availability

The data presented in this study are available in the Repository of the Medical University of Silesia [https://ppm.sum.edu.pl/info/phd/SUM6775dce44e7549b3be2373c36d40d74b/ (accessed on 20 January 2026)]. [Medical University of Silesia] [https://ppm.sum.edu.pl/info/phd/SUM6775dce44e7549b3be2373c36d40d74b/ (accessed on 20 January 2026)].

## Supplementary Materials

The following supporting information can be downloaded at: https://www.mdpi.com/article/10.3390/healthcare14070970/s1, Figure S1: Kaplan–Meier overall survival curves stratified by histopathological diagnosis. Figure S2: Kaplan–Meier overall survival curves for patients diagnosed with small-cell lung cancer (SCLC) stratified by age group. Figure S3: Kaplan–Meier overall survival curves for patients with non-small-cell lung cancer (NSCLC), stratified by age group. Figure S4: Kaplan–Meier overall survival curves for surgically treated patients, stratified by histopathological diagnosis. Figure S5: Kaplan–Meier overall survival curves for non-surgically treated patients, stratified by histopathological diagnosis. Figure S6: Kaplan–Meier overall survival curves for patients with small-cell lung cancer (SCLC), stratified by surgical versus non-surgical treatment. Figure S7: Kaplan–Meier overall survival curves for patients with small-cell lung cancer (SCLC) receiving first-line platinum derivatives combined with etoposide (PL+VEP), stratified by clinical stage. Figure S8: Kaplan–Meier overall survival curves for patients with small-cell lung cancer (SCLC) receiving platinum derivatives combined with etoposide (PL+VEP) without radiotherapy (RT), stratified by clinical stage. Figure S9: Kaplan–Meier overall survival curves for patients with small-cell lung cancer (SCLC) receiving platinum derivatives combined with etoposide (PL+VEP) and radiotherapy (RT), stratified by clinical stage. Figure S10: Kaplan–Meier overall survival curves for patients with small-cell lung cancer (SCLC) receiving a single cycle of platinum-based chemotherapy combined with etoposide and radiotherapy (1 CHT+RT), stratified by clinical stage. Figure S11: Kaplan–Meier overall survival curves for patients with small-cell lung cancer (SCLC) receiving exactly two cycles of platinum-based chemotherapy combined with etoposide and radiotherapy (2 CHT+RT), stratified by clinical stage. Figure S12: Kaplan–Meier overall survival curves for patients with small-cell lung cancer (SCLC) receiving three or more cycles of platinum-based chemotherapy combined with etoposide and radiotherapy (>2 CHT+RT), stratified by clinical stage. Figure S13: Kaplan–Meier overall survival curves for surgically treated patients with non-small-cell lung cancer (NSCLC), stratified by clinical stage. Figure S14: Kaplan–Meier overall survival curves for patients with non-small-cell lung cancer (NSCLC) receiving adjuvant chemotherapy, stratified by clinical stage. Figure S15: Kaplan–Meier overall survival curves for surgically treated patients with non-small-cell lung cancer (NSCLC), stratified by the first-line treatment regimen. Figure S16: Kaplan–Meier overall survival curves for surgically treated patients with non-small-cell lung cancer (NSCLC) receiving first-line platinum derivatives combined with vinorelbine (PL+NVB), stratified by clinical stage. Figure S17: Kaplan–Meier overall survival curves for surgically treated patients with non-small-cell lung cancer (NSCLC) receiving first-line platinum derivatives combined with gemcitabine (PL+GEM), stratified by clinical stage. Figure S18: Kaplan–Meier overall survival curves for patients with non-small-cell lung cancer (NSCLC) receiving adjuvant chemotherapy, stratified by the first-line treatment regimen. Figure S19: Kaplan–Meier overall survival curves for non-surgically treated patients with non-small-cell lung cancer (NSCLC), stratified by clinical stage. Figure S20: Kaplan–Meier overall survival curves for non-surgically treated patients with non-small-cell lung cancer (NSCLC) receiving platinum derivatives combined with vinorelbine (PL+NVB), stratified by clinical stage. Figure S21: Kaplan–Meier overall survival curves for non-surgically treated patients with non-small-cell lung cancer (NSCLC) receiving platinum derivatives combined with vinorelbine (PL+NVB), stratified by the number of cycles and treatment modality (chemotherapy vs. chemoradiotherapy). Figure S22: Kaplan–Meier overall survival curves for non-surgically treated patients with non-small-cell lung cancer (NSCLC) receiving platinum derivatives combined with gemcitabine (PL+GEM), stratified by clinical stage. Figure S23: Kaplan–Meier overall survival curves for non-surgically treated patients with non-small-cell lung cancer (NSCLC) receiving platinum derivatives combined with etoposide (PL+VEP), stratified by clinical stage. Figure S24: Kaplan–Meier overall survival curves for non-surgically treated patients with non-small-cell lung cancer (NSCLC) receiving platinum derivatives combined with etoposide (PL+VEP), stratified by the number of cycles and treatment modality. Figure S25: Kaplan–Meier overall survival curves for non-surgically treated patients with non-small-cell lung cancer (NSCLC) receiving gemcitabine (GEM) monotherapy, stratified by clinical stage. Figure S26: Kaplan–Meier overall survival curves for non-surgically treated patients with non-small-cell lung cancer (NSCLC) receiving gemcitabine (GEM) monotherapy, stratified by the number of cycles and the addition of radiotherapy. Figure S27: Kaplan–Meier overall survival curves for non-surgically treated patients with non-small-cell lung cancer (NSCLC) receiving vinorelbine (NVB) monotherapy, stratified by clinical stage. Figure S28: Kaplan–Meier overall survival curves for non-surgically treated patients with non-small-cell lung cancer (NSCLC) receiving vinorelbine (NVB) monotherapy, stratified by the number of cycles and treatment modality.

## Abbreviations

The following abbreviations are used in this manuscript:

NSCLC	Non-small-cell lung cancer
SCLC	Small-cell lung cancer
OS	Overall Survival
HR	Hazard Ratio
CI	Confidence Interval
CT	Computed Tomography
PET	Positron Emission Tomography
LDCT	Low-Dose Computed Tomography
KCO	Katowice Oncology Center (Katowickie Centrum Onkologii)
WHO	World Health Organization
PL	Platinum derivatives (cisplatin or carboplatin)
CHT	Chemotherapy
RT	Radiotherapy
GEM	Gemcitabine
NVB	Vinorelbine
VEP	Etoposide
ALI	Pemetrexed
NGS	Next-generation sequencing
EGFR	Epidermal Growth Factor Receptor
ALK	Anaplastic Lymphoma Kinase
