# Supplementary material for: Survival in Men Treated for Lung Cancer: A Single-Center Retrospective Cohort Study in Poland
Source: Healthcare (Basel). 2026 Apr 7;14(7):970. doi: 10.3390/healthcare14070970 (PMC13074180; doi:10.3390/healthcare14070970)
Supplement: Supplementary file 1 [file healthcare-14-00970-s001.zip › healthcare-4179964-supplementary.pdf]

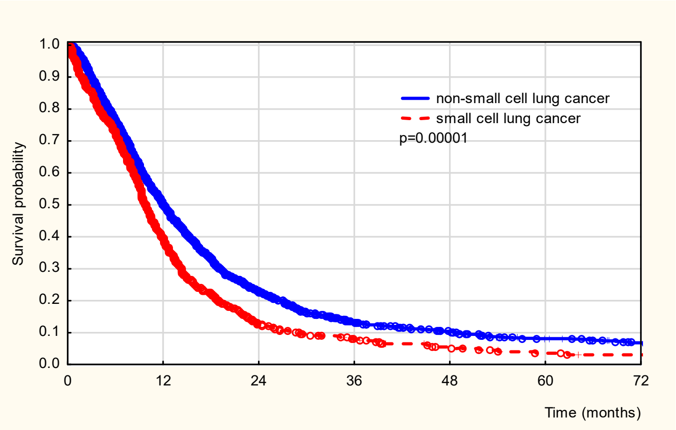

Figure S1. Kaplan–Meier overall survival curves stratified by histopathological diagnosis.

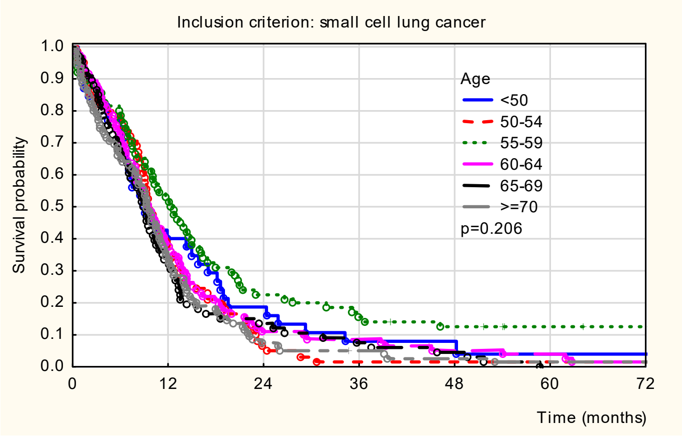

Figure S2. Kaplan–Meier overall survival curves for patients diagnosed with small cell lung cancer (SCLC) stratified by age group

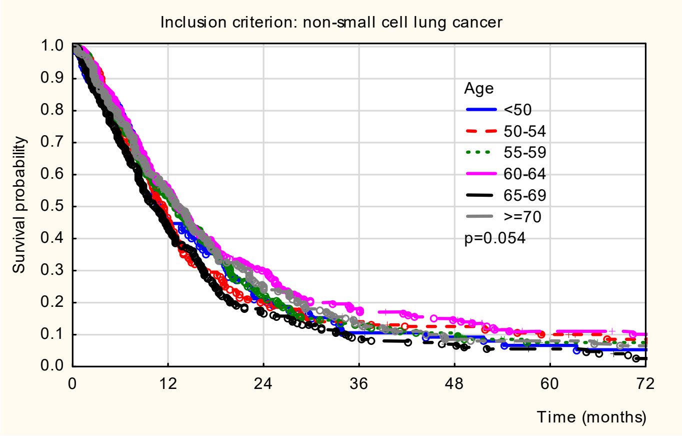

Figure S3. Kaplan–Meier overall survival curves for patients with non-small cell lung cancer (NSCLC), stratified by age group.

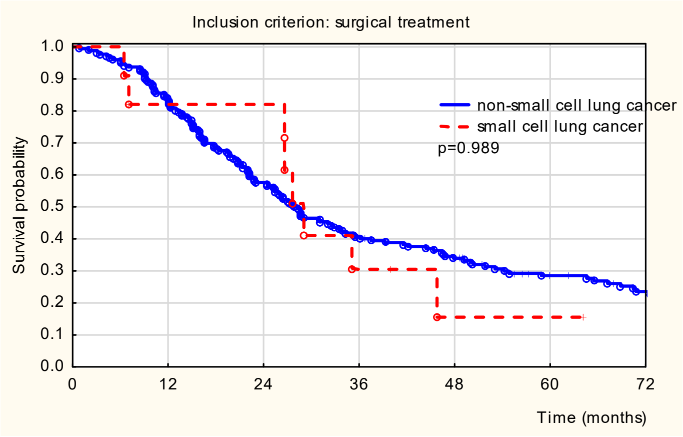

Figure S4. Kaplan–Meier overall survival curves for surgically treated patients, stratified by histopathological diagnosis.

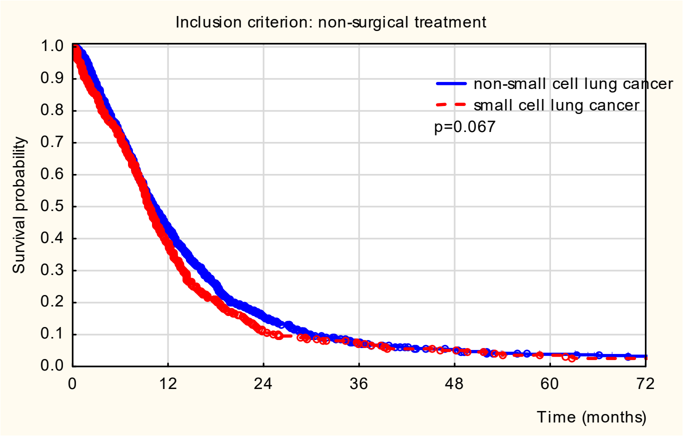

Figure S5. Kaplan–Meier overall survival curves for non-surgically treated patients, stratified by histopathological diagnosis.

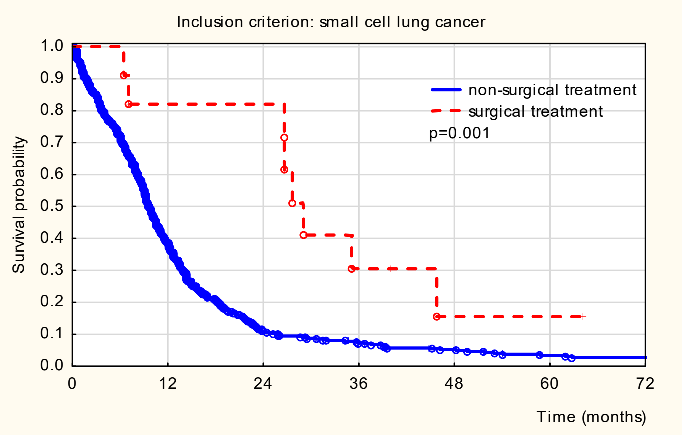

Figure S6. Kaplan–Meier overall survival curves for patients with small cell lung cancer (SCLC), stratified by surgical versus non-surgical treatment.

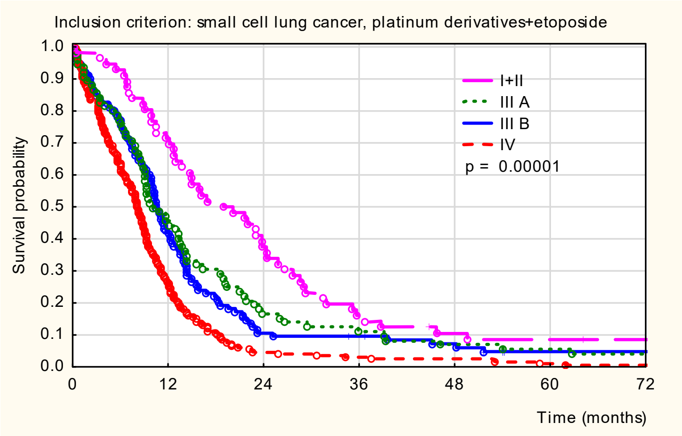

Figure S7. Kaplan–Meier overall survival curves for patients with small cell lung cancer (SCLC) receiving first-line platinum derivatives combined with etoposide (PL+VEP), stratified by clinical stage.

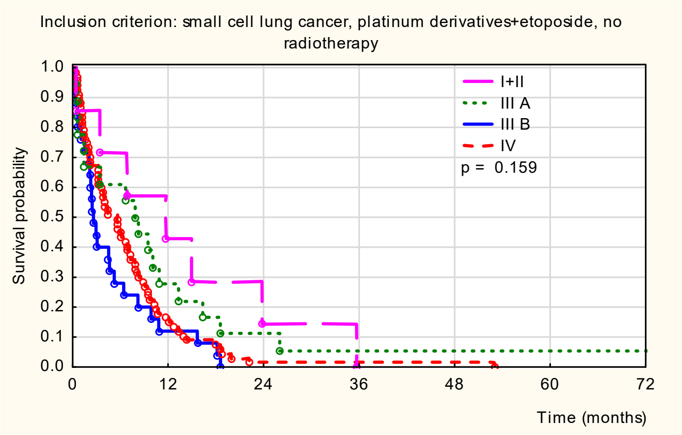

Figure S8. Kaplan–Meier overall survival curves for patients with small cell lung cancer (SCLC) receiving platinum derivatives combined with etoposide (PL+VEP) without radiotherapy (RT), stratified by clinical stage.

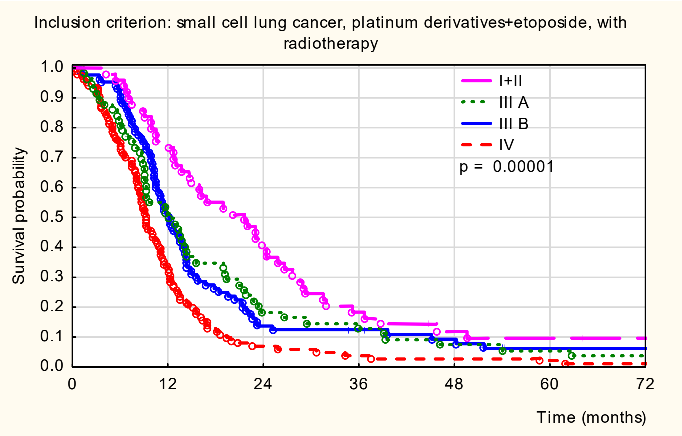

Figure S9. Kaplan–Meier overall survival curves for patients with small cell lung cancer (SCLC) receiving platinum derivatives combined with etoposide (PL+VEP) and radiotherapy (RT), stratified by clinical stage.

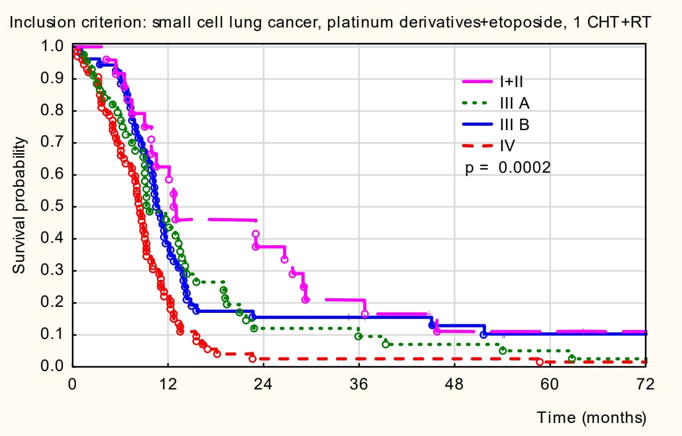

Figure S10. Kaplan–Meier overall survival curves for patients with small cell lung cancer (SCLC) receiving a single cycle of platinum-based chemotherapy combined with etoposide and radiotherapy (1 CHT+RT), stratified by clinical stage.

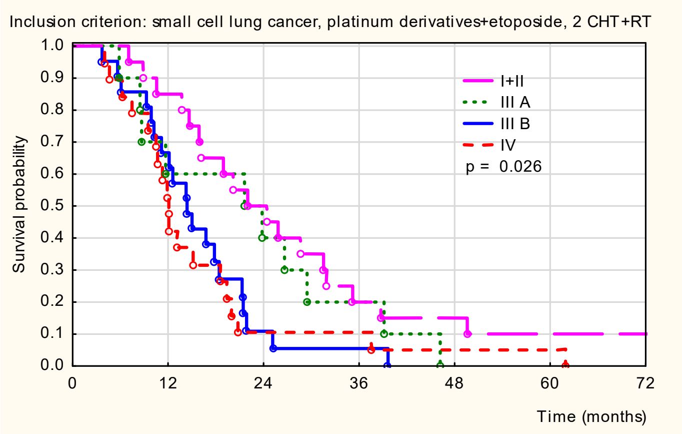

Figure S11. Kaplan–Meier overall survival curves for patients with small cell lung cancer (SCLC) receiving exactly two cycles of platinum-based chemotherapy combined with etoposide and radiotherapy (2 CHT+RT), stratified by clinical stage.

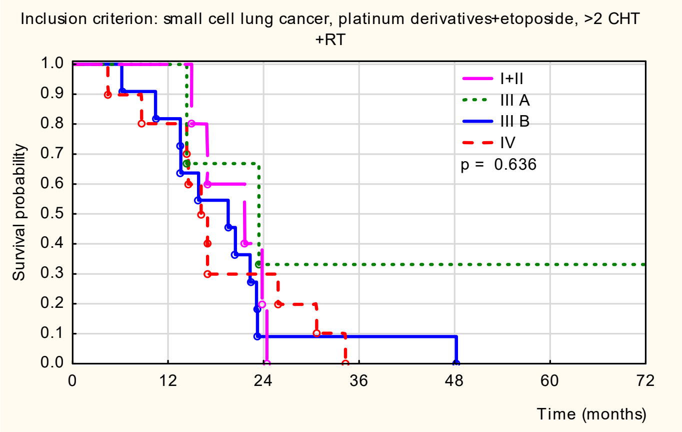

Figure S12. Kaplan–Meier overall survival curves for patients with small cell lung cancer (SCLC) receiving three or more cycles of platinum-based chemotherapy combined with etoposide and radiotherapy (>2 CHT+RT), stratified by clinical stage.

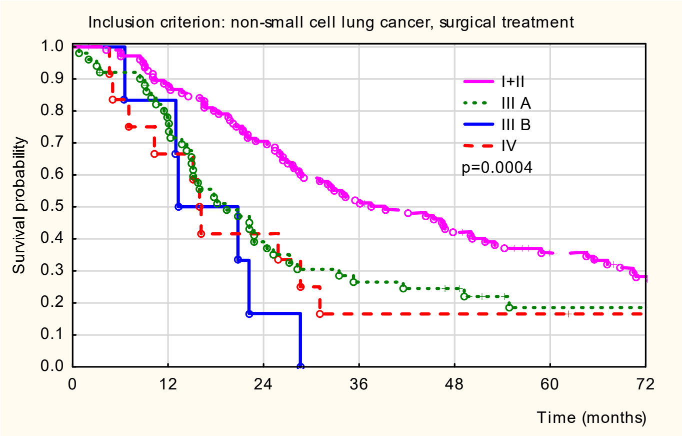

Figure S13. Kaplan–Meier overall survival curves for surgically treated patients with non-small cell lung cancer (NSCLC), stratified by clinical stage.

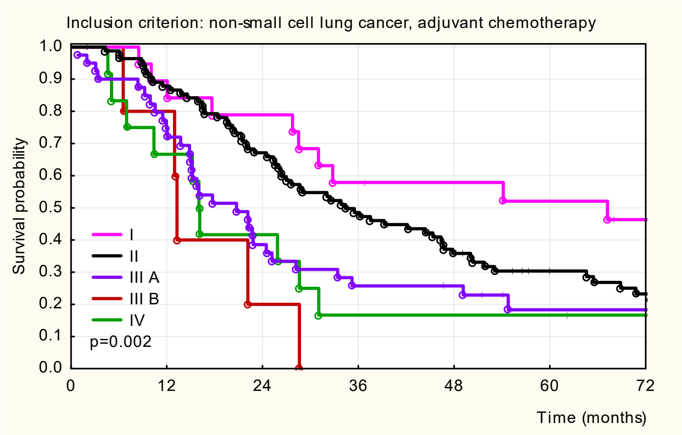

Figure S14. Kaplan–Meier overall survival curves for patients with non-small cell lung cancer (NSCLC) receiving adjuvant chemotherapy, stratified by clinical stage.

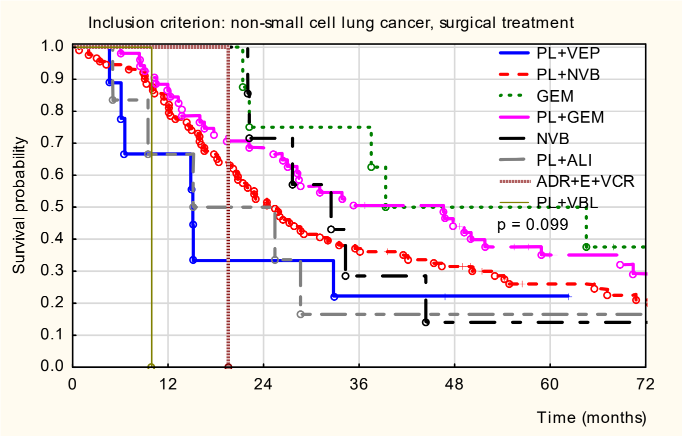

Figure S15. Kaplan–Meier overall survival curves for surgically treated patients with non-small cell lung cancer (NSCLC), stratified by the first-line treatment regimen.

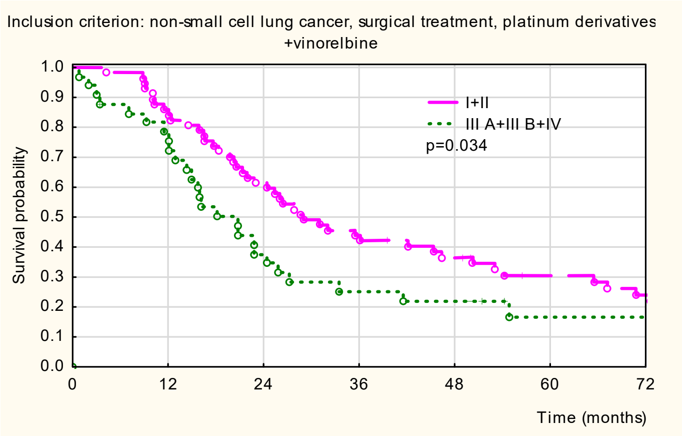

Figure S16. Kaplan–Meier overall survival curves for surgically treated patients with non-small cell lung cancer (NSCLC) receiving first-line platinum derivatives combined with vinorelbine (PL+NVB), stratified by clinical stage.

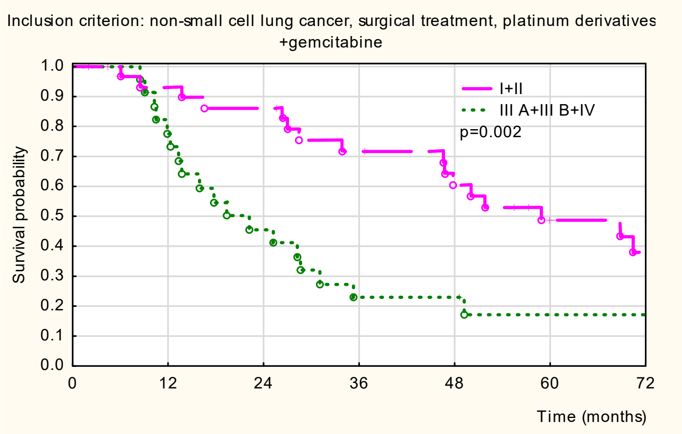

Figure S17. Kaplan–Meier overall survival curves for surgically treated patients with non-small cell lung cancer (NSCLC) receiving first-line platinum derivatives combined with gemcitabine (PL+GEM), stratified by clinical stage.

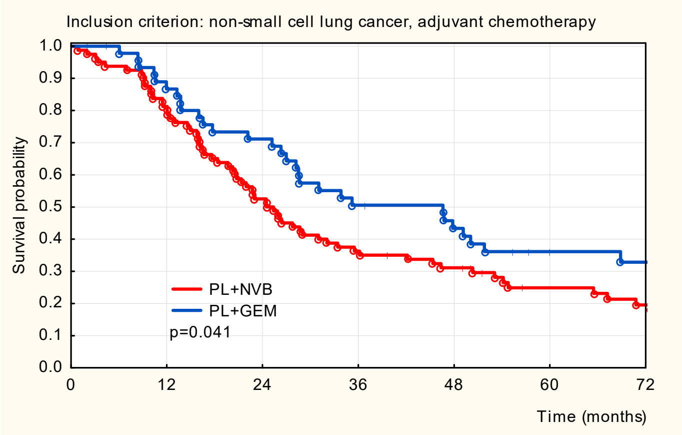

Figure S18. Kaplan–Meier overall survival curves for patients with non-small cell lung cancer (NSCLC) receiving adjuvant chemotherapy, stratified by the first-line treatment regimen.

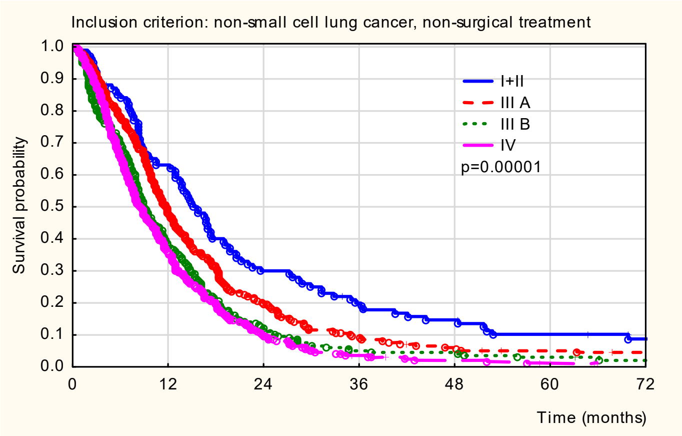

Figure S19. Kaplan–Meier overall survival curves for non-surgically treated patients with non-small cell lung cancer (NSCLC), stratified by clinical stage.

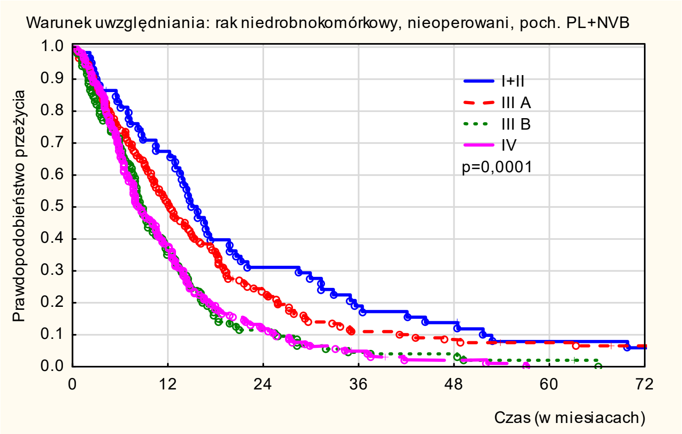

Figure S20. Kaplan–Meier overall survival curves for non-surgically treated patients with non-small cell lung cancer (NSCLC) receiving platinum derivatives combined with vinorelbine (PL+NVB), stratified by clinical stage.

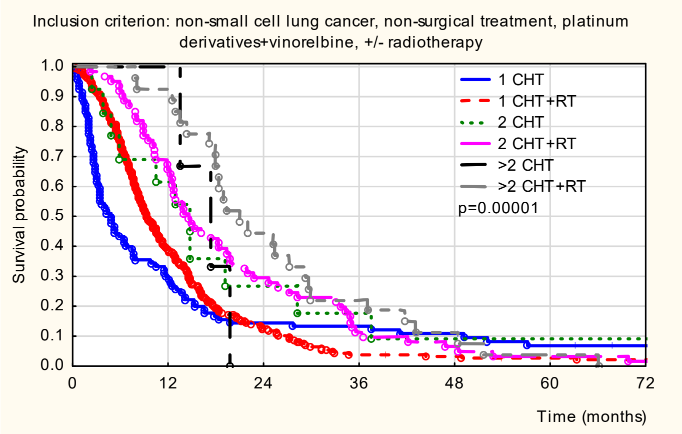

Figure S21. Kaplan–Meier overall survival curves for non-surgically treated patients with non-small cell lung cancer (NSCLC) receiving platinum derivatives combined with vinorelbine (PL+NVB), stratified by the number of cycles and treatment modality (chemotherapy vs. chemoradiotherapy).

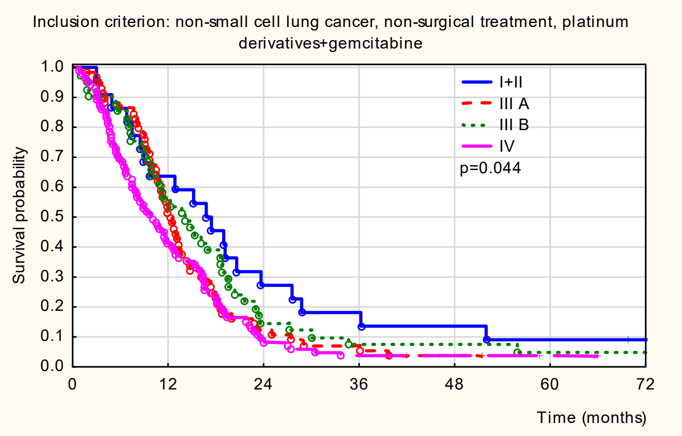

Figure S22. Kaplan–Meier overall survival curves for non-surgically treated patients with non-small cell lung cancer (NSCLC) receiving platinum derivatives combined with gemcitabine (PL+GEM), stratified by clinical stage.

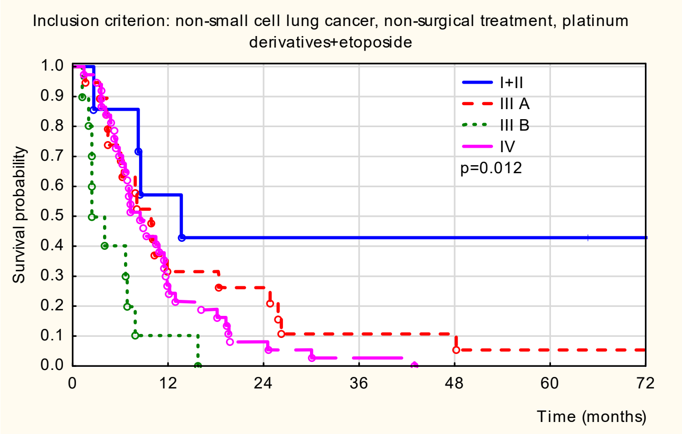

Figure S23. Kaplan–Meier overall survival curves for non-surgically treated patients with non-small cell lung cancer (NSCLC) receiving platinum derivatives combined with etoposide (PL+VEP), stratified by clinical stage.

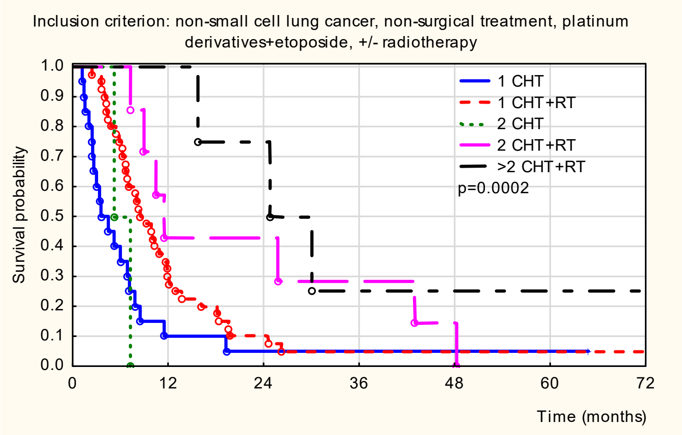

Figure S24. Kaplan–Meier overall survival curves for non-surgically treated patients with non-small cell lung cancer (NSCLC) receiving platinum derivatives combined with etoposide (PL+VEP), stratified by the number of cycles and treatment modality.

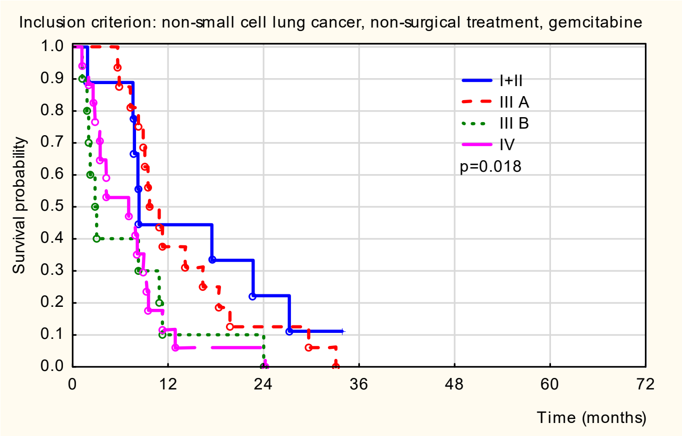

Figure S25. Kaplan–Meier overall survival curves for non-surgically treated patients with non-small cell lung cancer (NSCLC) receiving gemcitabine (GEM) monotherapy, stratified by clinical stage.

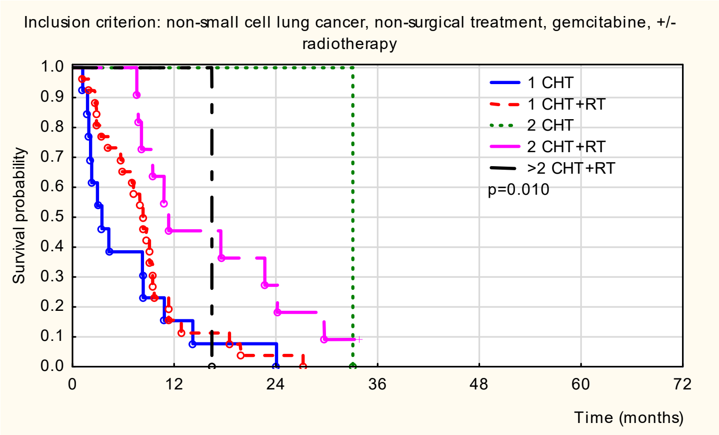

Figure S26. Kaplan–Meier overall survival curves for non-surgically treated patients with non-small cell lung cancer (NSCLC) receiving gemcitabine (GEM) monotherapy, stratified by the number of cycles and the addition of radiotherapy.

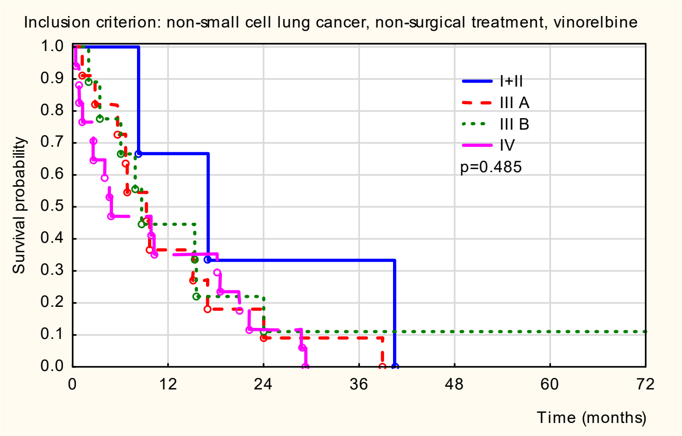

Figure S27. Kaplan–Meier overall survival curves for non-surgically treated patients with non-small cell lung cancer (NSCLC) receiving vinorelbine (NVB) monotherapy, stratified by clinical stage.

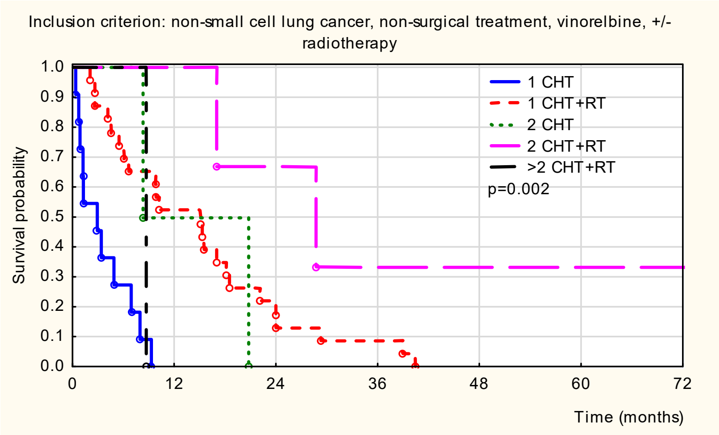

Figure S28. Kaplan–Meier overall survival curves for non-surgically treated patients with non-small cell lung cancer (NSCLC) receiving vinorelbine (NVB) monotherapy, stratified by the number of cycles and treatment modality.
